# Modulation of Re-initiation of Measles Virus Transcription at Intergenic Regions by P_XD_ to N_TAIL_ Binding Strength

**DOI:** 10.1371/journal.ppat.1006058

**Published:** 2016-12-09

**Authors:** Louis-Marie Bloyet, Joanna Brunel, Marion Dosnon, Véronique Hamon, Jenny Erales, Antoine Gruet, Carine Lazert, Christophe Bignon, Philippe Roche, Sonia Longhi, Denis Gerlier

**Affiliations:** 1 CIRI, International Center for Infectiology Research, Université de Lyon, Lyon, France; 2 INSERM, U1111, Lyon, France; 3 Ecole Normale Supérieure de Lyon, Lyon, France; 4 Université Claude Bernard Lyon 1, Centre International de Recherche en Infectiologie, Lyon, France; 5 CNRS, UMR5308, Lyon, France; 6 Aix-Marseille University, Architecture et Fonction des Macromolécules Biologiques (AFMB) UMR 7257, Marseille, France; 7 CNRS, AFMB UMR 7257, Marseille, France; 8 Aix Marseille University, Institut Paoli-Calmettes, Centre de Recherche en Cancérologie de Marseille (CRCM), Marseille, France; 9 CNRS, CRCM UMR 7258, Marseille, France; 10 INSERM, CRCM U1068, Marseille, France; Mayo Clinic, UNITED STATES

## Abstract

Measles virus (MeV) and all *Paramyxoviridae* members rely on a complex polymerase machinery to ensure viral transcription and replication. Their polymerase associates the phosphoprotein (P) and the L protein that is endowed with all necessary enzymatic activities. To be processive, the polymerase uses as template a nucleocapsid made of genomic RNA entirely wrapped into a continuous oligomer of the nucleoprotein (N). The polymerase enters the nucleocapsid at the 3’end of the genome where are located the promoters for transcription and replication. Transcription of the six genes occurs sequentially. This implies ending and re-initiating mRNA synthesis at each intergenic region (IGR). We explored here to which extent the binding of the X domain of P (XD) to the C-terminal region of the N protein (N_TAIL_) is involved in maintaining the P/L complex anchored to the nucleocapsid template during the sequential transcription. Amino acid substitutions introduced in the XD-binding site on N_TAIL_ resulted in a wide range of binding affinities as determined by combining protein complementation assays in *E*. *coli* and human cells and isothermal titration calorimetry. Molecular dynamics simulations revealed that XD binding to N_TAIL_ involves a complex network of hydrogen bonds, the disruption of which by two individual amino acid substitutions markedly reduced the binding affinity. Using a newly designed, highly sensitive dual-luciferase reporter minigenome assay, the efficiency of re-initiation through the five measles virus IGRs was found to correlate with N_TAIL_/XD K_D_. Correlatively, P transcript accumulation rate and F/N transcript ratios from recombinant viruses expressing N variants were also found to correlate with the N_TAIL_ to XD binding strength. Altogether, our data support a key role for XD binding to N_TAIL_ in maintaining proper anchor of the P/L complex thereby ensuring transcription re-initiation at each intergenic region.

## Author Summary

Three proteins, the polymerase L, the phosphoprotein P and the nucleoprotein N, interplay to ensure transcription and replication of measles virus, a member of the *Paramyxoviridae* family. A regular array of nucleoprotein shields the viral genomic RNA. The resulting nucleocapsid constitutes the template of RNA synthesis used by the polymerase complex made of L and P, with the latter ensuring L anchoring onto the nucleocapsid. We herein report a correlation between the binding affinity of the C-terminal X domain of P (XD) and the intrinsically disordered C-terminal tail of N (N_TAIL_), the ability to reinitiate the transcription at the intergenic regions and the accumulation rate of viral transcripts from recombinant viruses. We therefore propose that the N_TAIL_/XD interaction contributes to maintaining the polymerase complex anchored onto the nucleocapsid while ending the upstream transcript and re-initiating the downstream transcript at every intergenic region. As such, the N_TAIL_/XD interaction strength must be controlled so as to keep the viral transcription gradient within an optimal efficiency window. The conservation of this mode of interaction between the viral P and N proteins in many members of the *Paramyxoviridae* family reflects one of the major evolution constraints to which their polymerase machinery is subjected.

## Introduction

Measles virus (MeV), a member of the *Morbillivirus* genus, belongs to the *Paramyxoviridae* family of the *Mononegavirales* order [1]. These viruses possess a non-segmented RNA genome of negative polarity that is encapsidated by the nucleoprotein (N) to form a helical nucleocapsid. Not only does N protect viral RNA from degradation and/or formation of viral dsRNA, but it also renders the latter competent for transcription and replication. Indeed, the viral polymerase cannot processively transcribe nor replicate RNA unless the viral genome is encapsidated by the N protein within a helical nucleocapsid [2,3]. Transcription and replication are ensured by the RNA-dependent RNA polymerase complex made of the large protein (L) and the phosphoprotein (P), with P serving as an essential tethering factor between L and the nucleocapsid. The complex made of RNA and of the N, P and L proteins constitutes the replication machinery. In order to perform messenger RNA synthesis, the polymerase has not only to bind to the 3’ transcription promoter, but also to re-initiate the transcription of downstream genes upon crossing each intergenic region (IGR). Following polyadenylation, which serves as gene end (GE) signal, the polymerase proceeds over three nucleotides (3’-GAA-5’ or 3’-GCA-5’) without transcribing them and then restarts transcription upon recognition of a downstream gene start (GS) signal.

Within infected cells, N is found in a soluble, monomeric form (referred to as N^0^) and in a nucleocapsid assembled form [4]. Following synthesis, the N protein requires chaperoning by the P protein so as to be maintained in a soluble and monomeric form. The P N-terminal region (P_NT_) binds to the neosynthesized N protein thereby simultaneously preventing its illegitimate self-assembly and yielding a soluble N^0^P complex the structure of which have been characterised for MeV [5] as well as for four other members of the *Mononegavirales* order [6,7,8,9]. N^0^P is used as the substrate for the encapsidation of the nascent genomic RNA chain during replication [10], (see also [4,11,12,13] for reviews on transcription and replication). In its assembled homopolymeric form or nucleocapsid, N also makes complexes with either isolated P or P bound to L, with all these interactions being essential for RNA synthesis by the viral polymerase [14,15,16]. Throughout the *Mononegavirales* order, P and P+L binding to the nucleocapsid is mediated by interaction of the C-terminal region of P with either the C-terminal tail of N (*Paramyxoviridae* members), or to the N-terminal globular moiety (or core) of N (see [11,17] for review).

**Fig 1:**
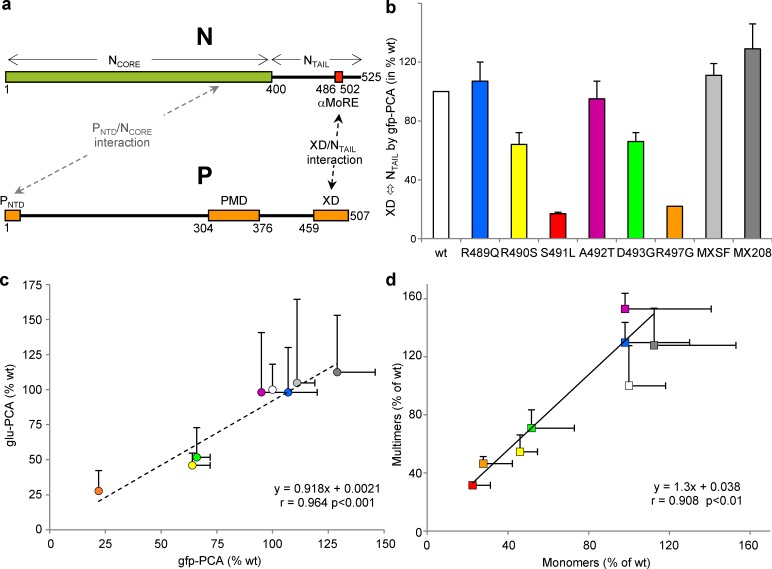
Binding affinity of the NTAIL variants as evaluated by gfp- and glu-PCA. **(a)** Representation of N and P proteins. **(b)** Relative binding strength between N_TAIL_ variants and XD as assessed by gfp-PCA. **(c)** Correlation between binding strengths as inferred from gfp-PCA and as inferred from glu-PCA. **(d)** Correlation between binding strengths as obtained from monomeric glu-PCA and from multimeric (i.e. P_MD-XD_ against N) glu-PCA. Shown are means and standard deviations (SD) from three independent experiments performed in triplicate. The color code used for symbols is the same in panel b-d. The color code for N variants adopted in panel **(b)** will be used throughout all figures.

The MeV N protein consists of a structured N-terminal moiety (N_CORE_, aa 1–400), and a C-terminal domain (N_TAIL_, aa 401–525) [18,19] that is intrinsically disordered, i.e. it lacks highly populated secondary and tertiary structure under physiological conditions of pH and salinity in the absence of a partner (for a recent review on intrinsically disordered proteins see [20]). While N_CORE_ contains all the regions necessary for self-assembly and RNA-binding [10,21,22] and a binding site for an α-MoRE located at the N terminus of the P protein, N_TAIL_ is responsible for interaction with the C-terminal X domain (XD, aa 459–507) of P [11,18,21,23,24,25,26,27] (**Fig 1A**).

N_TAIL_ binding to XD triggers α-helical folding within a molecular recognition element [28,29] of α-helical nature (α-MoRE, aa 486–502) located within one (Box2, aa 489–506) out of three conserved N_TAIL_ regions [18,24,27,30,31,32,33,34,35,36,37]. XD-induced α-helical folding of N_TAIL_ is not a feature unique to MeV, being also conserved within the *Paramyxoviridae* family [38,39,40,41,42,43]. XD consists of a triple α-helical bundle [27,34,44], and binding to the α-MoRE leads to a pseudo-four-helix arrangement that mainly relies on hydrophobic contacts [18,27,44]. The α-MoRE of N_TAIL_ is partly preconfigured as an α-helix prior to binding to XD [31,32,35,37,45] and adopts an equilibrium between a fully unfolded form and four partly helical conformers [37]. In spite of this partial pre-configuration, N_TAIL_ folds according to a “folding after binding mechanism” [45,46]. Previous mutational studies showed that Box2 is poorly evolvable in terms of its binding abilities towards XD, in that amino acid substitutions therein introduced lead to a dramatic drop in the binding strength, as judged from a protein complementation assay (PCA) based on split-GFP reassembly (gfp-PCA) [47]. In particular, substitutions within the N-terminal region of Box2 (aa 489–493) and at position 497 were found to lead to the most dramatic drops in the interaction strength [47].

In the context of the viral nucleocapsid, N_TAIL_ points towards the interior of the latter and then extrafiltrates through the interstitial space between N_CORE_ moieties, with the first 50 residues (aa 401–450) being conformationally restricted due to their location between successive turns of the nucleocapsid [37]. The N_TAIL_ region spanning residues 451–525 and encompassing the α-MoRE is, by contrast, exposed at the surface of the viral nucleocapsid and thus accessible to the viral polymerase. Binding of XD to N_TAIL_ has been proposed to ensure and/or contribute to the recruitment of the viral P/L polymerase complex onto the nucleocapsid template. However, its precise function has remained enigmatic so far with reports of apparent conflicting observations. From the analysis of four N_TAIL_ variants it was concluded that the accumulation rate of primary transcripts is rather insensitive to a drop in the apparent XD to N_TAIL_ affinity [26], while an XD variant showing a 1.7 times stronger interaction with N_TAIL_ was associated with a 1.7-fold reduction in the accumulation rate of viral transcripts [48]. Furthermore, deletions studies of N_TAIL_ have indicated that the interaction between XD and N_TAIL_ may be dispensable for transcription and replication [49].

In the present work, we further investigate the molecular mechanisms by which substitutions in critical positions of N_TAIL_ previously identified by a random approach [47] affect the viral polymerase activity. We did so by combining biochemical studies and molecular dynamics (MD) simulations on one hand with functional studies that made use of minigenomes and recombinant viruses on the other hand. Results identify positions 491 and 497 as the most critical in terms of both binding affinities and functional impact. In addition, thanks to the availability of a newly conceived minigenome made of two luciferase reporter genes, with the second one being conditionally expressed via RNA edition of its transcript by the viral polymerase, we could quantify the efficiency of transcription re-initiation after polymerase scanning through each of the five IGRs of MeV genome and on an elongated un-transcribed IGR (UTIGR). A low N_TAIL_-XD affinity was found to be associated to a reduced ability of N to support expression of luciferase from the second gene. Furthermore, in infected cells, the accumulation rate of primary transcripts and transcript ratios were found to correlate with the equilibrium dissociation constant (K_D_) of the N_TAIL_/XD pair. Altogether obtained data argue for a key role of the N_TAIL_/XD interaction in transcription re-initiation at each intergenic region.

## Results

### Binding strengths of N_TAIL_ variants from two protein complementation assays

In a previous random mutagenesis study that made use of a PCA based on split-GFP re-assembly (gfp-PCA) [47], we identified N_TAIL_ variants that either decrease or increase the interaction strength towards XD [47]. Variant MX208, which bears the D437V, P485L and L524R substitutions that are all located outside the α-MoRE, is an example of the latter group. We previously reported the generation and assessment of binding properties by gfp-PCA of six single-site variants (R489Q, R490S, S491L, A492T, D493G and R497G) bearing each a unique substitution within the α-MoRE [47]. Here, we additionally designed and generated the MXSF variant, which bears D437V, R439S, P456S and P485L substitutions that are all found in variants displaying an increased fluorescence [47]. Gfp-PCA in *E*. *coli* showed that the binding strength of these variants towards XD is scattered over a wide range, with the S491L and R497G variants showing the lowest interaction and with variant MXSF displaying interaction strength only moderately higher than *wt* N_TAIL_ (**Fig 1B**). Incidentally, this latter finding indicates that the effects of the substitutions are not cumulative.

We then sought at assessing to which extent results afforded by the split-GFP assay in *E*. *coli* cells reflect N_TAIL_/XD binding occurring in the natural host cells of MeV. To this end, the interaction between XD and N_TAIL_ variants was measured using the split-luciferase reassembly assay [50]. This technique is based on the same principle as the split-GFP reassembly assay. The reporter (i.e. *Gaussia princeps* luciferase) and the measured parameter (luminescence) are however different, and the assay is performed in human cells. Moreover, contrary to the split-GFP reassembly assay where reporter reassembly is irreversible, in the split-luciferase assay (glu-PCA), association of the two luciferase fragments is reversible. As such, while the measured parameter in the former assay is dominated by the *k*_*on*_, the measured parameter in the latter assay does reflect the equilibrium between a *k*_on_ and a *k*_off_ and hence a true K_D_. A significant correlation was obtained between the two PCA methods (**Fig 1C**), a finding that provides additional support for the significance of the observed differences in binding strength among variants. Furthermore, a significant correlation was also observed when comparing binding strengths as obtained using monomeric constructs (i.e. N_TAIL_/XD) and binding strengths obtained using their natural multimeric counterparts, i.e. P multimerization domain (PMD)-XD (P303-507) and full-length mutated N protein constructs (**Fig 1D**). The rationale for using P multimeric constructs devoid of the N-terminal region (P_NT_) was to eliminate the binding site to N_CORE_ located within P_NT_ and involved in P chaperoning of N protein to form N^0^P complexes [5] (see **Fig 1A** for depicting scheme). Importantly, all N variants accumulated in cells in similar amounts (**S1 Fig)** indicating that variations in the level of reconstituted Gaussia luciferase likely reflects variations in N_TAIL_ to XD binding strength.

### Generation, purification and characterization of the single-site Box2 N_TAIL_ variants

**Fig 2:**
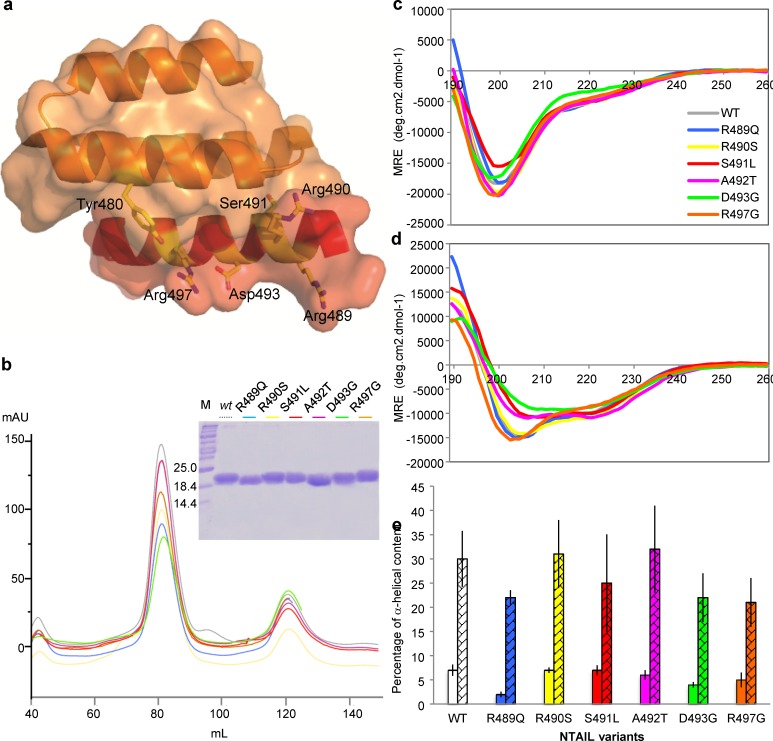
**Secondary structure content of NTAIL variants (a)** Crystal structure of the chimera between XD (orange) and the α-MoRE of N_TAIL_ (red) (PDB code 1T6O) [44]. The side chains of residues mutated in the N_TAIL_ variants investigated in this study are shown in sticks with atom type colours. **(b)** SEC elution profile of the purified N_TAIL_ variants. The differences in the peak heights reflect differences in the total amounts of protein loaded onto the SEC column. Inset. Coomassie blue staining of an 18% SDS-PAGE analysis of the purified N_TAIL_ variants. M: molecular mass markers. Color code is shown above the Coomassie blue staining picture with dotted grey line for wt N. **(c)** Far-UV CD spectra of the purified N_TAIL_ variants at 0.1 mg/ml in 10 mM sodium phosphate buffer at pH 7 either in the absence **(c)** or in the presence of 20% TFE **(d)**. Shown are the average CD spectra as obtained from three different protein samples. **(e)** α-helical content of N_TAIL_ variants in the absence (plain bars) or presence (hatched bars) of 20% TFE, as obtained by deconvoluting CD spectra using the Dichroweb server.

In order to characterize Box2 variants (**Fig 2A**) at the biochemical level, we expressed and purified six α-MoRE variants of N_TAIL_ as N-terminally hexahistidine tagged proteins. All N_TAIL_ variants were purified to homogeneity from the soluble fraction of the bacterial lysate through immobilized metal affinity chromatography (IMAC) followed by size exclusion chromatography (SEC) (**Fig 2B**). The identity of all purified proteins were checked and confirmed by mass spectrometry. Even if their molecular mass is ~16 kDa, they all migrate on a denaturing gel with an apparent molecular mass of approximately 20 kDa (**Fig 2B, inset**). This aberrant electrophoretic migration has been systematically observed for all N_TAIL_ variants reported so far [26,30,31,32,46] including *wt* N_TAIL_ [19]. This anomalous migration is frequently observed in IDPs and is due to a high content in acidic residues [51] and/or a large extension in solution [43].

All N_TAIL_ variants, including *wt* N_TAIL,_ have the same SEC elution profile (**Fig 2B**). In particular, they are all eluted with an apparent molecular mass higher than expected and typical of a premolten globule (PMG) state [52], as already observed in the case of *wt* N_TAIL_ [19]. Thus, the amino acid substitution(s) causes little (if any) effect on the hydrodynamic volume sampled by the protein.

Analysis of the secondary structure content of the N_TAIL_ variants by far-UV circular dichroism (CD) shows they are all disordered, as judged from their markedly negative ellipticity at 200 nm (**Fig 2C**). In addition, they are all similarly able to gain α-helicity in the presence of 20% 2,2,2 trifluoroethanol (TFE) (**Fig 2D**), as already observed for *wt* N_TAIL_ [19]. All variants have an estimated α-helical content similar (within the error bar) to that of *wt* N_TAIL_, with the only exception of variant R489Q that exhibits a lower α-helicity both in the absence and in the presence of TFE **(Fig 2E**). Thus, most of the amino acid substitutions cause little (if any) effect on the overall secondary structure content and folding abilities of N_TAIL_.

### Assessment of binding affinities of single-site Box2 N_TAIL_ variants towards XD by ITC

**Fig 3:**
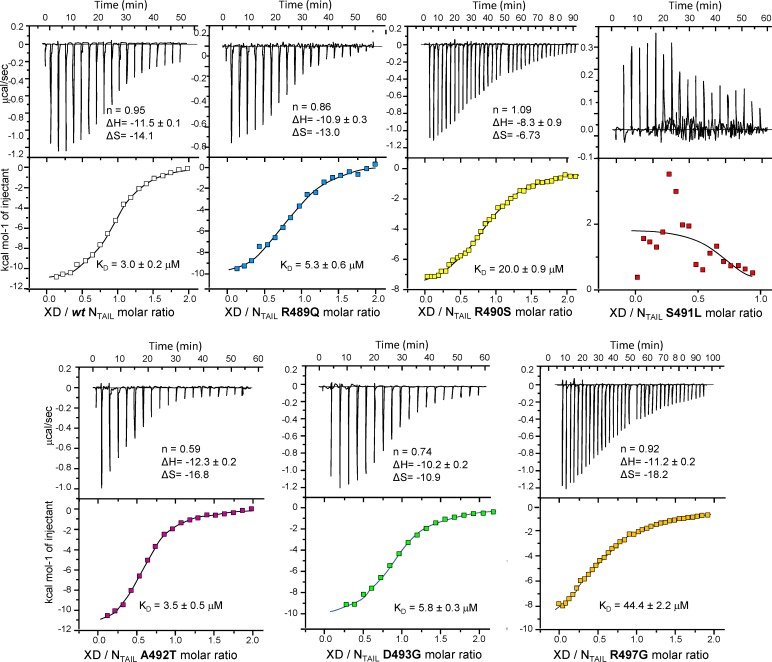
Binding parameters of the N_TAIL_ variants towards XD as obtained by ITC. Data are representative of at least two independent experiments. The derived equilibrium dissociation constants (K_D_), the stoichiometry number (n), the binding enthalpy ΔH (kcal mol^-1^), and the binding entropy ΔS (cal mol^-1^ deg^-1^) are shown. Shown are the curves obtained using the following concentrations of N_TAIL_ in the microcalorimeter cell and of XD in the microsyringe: wt N_TAIL_/XD: 50 μM/500 μM; N_TAIL_ R489Q/XD: 50 μM/500 μM; N_TAIL_ R490S/XD: 150 μM/854 μM; N_TAIL_ S491L/XD: 160 μM/800 μM; N_TAIL_ A492T/XD: 50 μM/500 μM; N_TAIL_ D493G/XD: 25 μM/600 μM; N_TAIL_ R497G/XD: 150 μM/955 μM. Graphs shown at the bottom of each panel correspond to integrated and corrected ITC data fitted to a single set of sites model. Note that for the binding reactions characterized by K_D_ values in the tens of micromolar range, it is difficult to obtain the first plateau because the necessary concentrations are too high. Consequently, it should be kept in mind that the actual errors may be larger than those estimated by the fit.

The binding abilities of the N_TAIL_ variants, including *wt* N_TAIL_, were assessed using isothermal titration calorimetry (ITC). To this end, the purified N_TAIL_ proteins were loaded into the sample cell of an ITC200 microcalorimeter and titrated with *wt* XD. For each variant, two independent experiments were carried out. **Fig 3** shows, for each variant, one representative ITC curve along with the relevant binding parameters. The XD/N_TAIL_ molar ratios achieved at the end of the titration were 2.0 (*wt*, R489Q, A492T, D493G), 2.5 (R490S) or 3.0 (R497G) (**Fig 3**). The data, following integration and correction for the heats of dilution, were fitted with a standard model allowing for a set of independent and equivalent binding sites.

Consistent with the unfavorable entropic contribution associated to the disorder-to-order transition that takes place upon N_TAIL_ binding to XD, whenever binding parameters could be determined, they revealed a decrease in entropy, with a ΔS ranging from -13 to -29.5 cal mol^-1^ deg^-1^ (**Fig 3**). Binding reactions were all found to be enthalpy-driven, with ΔH values in the same order of magnitude and ranging from -10.9 to -14.5 kcal/mol (**Fig 3**). The estimates for the model parameters of the *wt* N_TAIL_/XD pair were found to be in very good agreement with those recently reported [46]. The estimates for binding parameters of variants R489Q, A492T and D493G yielded equilibrium dissociation constant (K_D_) very close to that observed for *wt* N_TAIL_, indicating that these substitutions poorly affect the interaction (**Fig 3**). On the other hand, the R490S substitution resulted in a 7-fold decrease in the binding affinity (K_D_ of 20 μM). The decrease in affinity was even further pronounced (K_D_ of 44 μM) in the case of the R497G variant, although the interaction remained measurable (**Fig 3**). In the case of the S491L variant the interaction strength was below the ITC detection limit and thus K_D_ could not be estimated (**Fig 3**).

The *n* values for the A492T/XD and D493G/XD binding pairs were found to deviate from unit, a behaviour that is not unusual and that has been already observed with single-site tryptophan variants [46] and that may arise from relatively poorly defined baselines. In light of all the numerous previous studies [18,19,27,30,31,32,33,34,35,36,37] showing that N_TAIL_ and XD form a 1:1 complex, these deviations were not taken to be significant.

**Fig 4:**
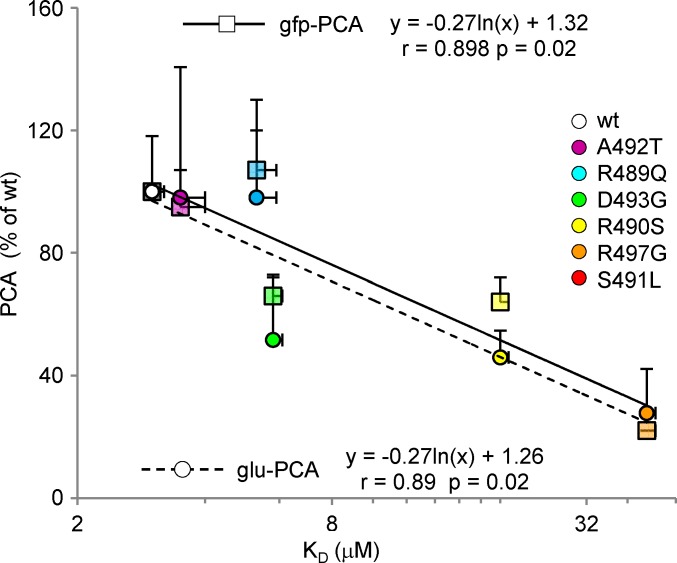
Correlation between binding strengths, as inferred from either gfp-PCA of glu-PCA, and binding affinities derived from ITC. In both cases a statistically significant correlation was observed between fluorescence or luminescence values and the K_D_ (p = 0.02 in both cases). Shown are the mean values and SD as obtained from two (ITC) or three (PCAs) independent experiments performed in triplicate.

We next focused on how binding affinities obtained by ITC correlate with binding strengths inferred from split-GFP and split-luciferase reassembly assays. In fact, although it has already been established that the higher the fluorescence the higher the interaction strength [53], no attempts were done at establishing which type of relationship exists between K_D_ values and fluorescence or luminescence values. As shown in **Fig 4**, we found a significant correlation between fluorescence or luminescence values obtained by gfp-PCA [47] and glu-PCA and the *ln* of K_D_ values (p = 0.02 in both cases). Although this finding needs to be confirmed on a larger set of data points, it lays the basis for the possibility of inferring K_D_ values directly from fluorescence or luminescence values.

Notably, if the results obtained by gfp-PCA (see **Fig 1B**) pointed out similarly low interaction strengths for variants S491L and R497G, ITC studies yielded a different profile. Indeed, while no interaction could be effectively detected for the S491L/XD pair, the K_D_ could be measured for variant R497G (see **Fig 3**). Using the empirically determined equation relating luminescence and K_D_ values (**Fig 4**), the K_D_ of the S491L/XD pair was estimated to be 85 ± 33 μM, a value consistent with our inability to detect the interaction by ITC. Indeed, an interaction characterized by a K_D_ of approximately 100 μM could escape detection unless extremely high (and hardly achievable) protein concentrations are used (typically 1 mM N_TAIL_/10 mM XD) [54].

Altogether, obtained results confirmed that not all Box2 residues are equivalent in terms of their role in N_TAIL_/XD complex formation. In particular, while substitutions at positions 489, 490, 492 and 493 have a slight to moderate impact, substitutions at positions 497 and 491 drastically affect complex formation without having a strong impact on the overall α-helicity. The role of Box2 residues in complex formation follows the order 491>497>490, reflecting either the orientation of side chains towards the partner (residues 490 and 491) or involvement in stabilizing interactions with XD residue Tyr480 in spite of solvent exposure (residue 497), as already proposed (**Fig 2A**) [47].

### Molecular dynamic simulations of wt, S491L and R497G variants in complex with XD reveal a crucial hydrogen bonding network

In order to further investigate the mechanisms by which residues Ser491 and Arg497 stabilize the N_TAIL_/XD complex, we performed MD simulations in aqueous solvent using the CHARMM force field [55]. MD simulations were carried out starting from the X-ray structure of the XD/α-MoRE complex [44] or from the *in silico* generated XD/α-MoRE S491L and XD/α-MoRE R497G models. In the case of the S491L variant, the three most favorable orientations of the side chains were generated. We first assessed the dynamical stability of the complexes. For this purpose, we analyzed the root-mean square deviation (RMSD) of the Cα atoms with respect to the initial structure as a function of time for the three complexes (i.e. *wt*, S491L and R497G) (**S1 Table**). The RMSD values showed very little variations between the different constructs during the time course of the 50 ns simulations (**S1 Table**). The average RMSD for XD and for the α-MoRE were approximately 0.8 and 0.5 Å, respectively, indicating structural stability of each domain during the simulations (**S1 Table**). The relative orientation of the α-MoRE compared to XD was also assessed and revealed slightly higher average RMSD values for the two variants due to small local rearrangements of the structures to adapt to the substitutions. However, RMSD fluctuations were in the same order of magnitude. Secondary structure analyses of *wt* and mutated complexes confirmed that all α-helices are conserved during the whole trajectories. Overall, the different systems were stable during the whole simulation. Since the orientation of the side-chain of L491 showed no impact on the behavior of the complex during the MD simulation, only one conformer was selected in the rest of the study.

Although the association between XD and N_TAIL_ is essentially driven by hydrophobic contacts, the two partners also interact through hydrogen bonds that are thus expected to play a role in the binding affinity. Two intramolecular hydrogen-bond interactions are present in the crystallographic structure of the complex (**Table 1)**. These interactions involve the side-chain of N_TAIL_ residue Ser491 and side-chain of Asp493 and main-chain of Lys489 from XD. These interactions are preserved in the simulations of the *wt* and R497G complex (**Table 1 and S2 Fig**). Due to the absence of the polar OH group in leucine, hydrogen bonds involving the OH group of Ser491 were lost in the simulations of the S491L complex. Three additional hydrogen bonds that are not present in the X-ray structure were observed in the MD trajectories of the *wt* complex (**Table 1 and S2 Fig**). Two of them involve the side-chain of Asp487 from XD and side-chains of either Arg490 or Arg497 of N_TAIL_. Only the former was also observed in the simulations of both variants (**Table 1 and S2 Fig**). The third one was formed between the side-chains of Lys489 from XD and Asp487 from N_TAIL_ and was detected in the simulation of the three complexes with the two variants exhibiting even a higher frequency (**Table 1 and S2 Fig**). In addition, a water-mediated hydrogen bond could be identified between the side-chains of Tyr480 of XD and Arg497 of N_TAIL_, 55 and 41 percent of the time in the *wt* and S491L complex, respectively. This interaction was not maintained with the same water molecule throughout simulation. However, when a water molecule moved away from this site it was almost immediately replaced by another water molecule. This interaction could not occur in the R497G complex and was not compensated by another interaction. The presence of this water-mediated interaction correlates with the stabilization of the aromatic ring of Tyr480. The side-chain of Tyr480 was found in almost only one conformation corresponding to a χ2 angle (CA-CB-CG-CD) of approximately -130° in both *wt* and S491L complexes, whereas in the R497G complex, the ring oscillates between 2 conformations (50 and -130°) corresponding to a 180° rotation. Although the position of this water molecule in the crystal structure cannot be estimated with precision because the molecule is poorly defined in the electron density, the fact that a water molecule is systematically observed at this position during the simulation argues for its critical role in stabilizing the Arg497-Tyr480 interaction. That water molecules can play crucial roles in stabilizing protein-protein interactions has been widely documented [56].

**Table 1:** Frequency of major intermolecular hydrogen bonds during the 50 ns MD trajectories of XD/α-MoRE complexes (wt and mutated). MD shows the key role of N_TAIL_ S491 for stable binding to XD.

XD			N_TAIL_			Hydrogen bond frequency (% over 50 ns)^1^		
aa	Main/ Side chain atom	Acceptor/Donor	aa	Main/ Side chain atom	Acceptor/Donor	wt	R497G	S491L
**D487**	S	A	**R490**	S	D	**44.2**	**38.7**	**37.1**
**D487**	S	A	**R497**	S	D	**12.5**		
**K489**	S	D	**D487**	S	A	**12.0**	**18.8**	**24.4**
**K489**	M	D	**S491**	S	A	**53.8** ^**2**^	**28.1** ^**2**^	
**D493**	S	A	**S491**	S	D	**41.6** ^**2**^	**42.1** ^**2**^	

^1^ Transiently observed hydrogen bonds during less than 1 ns are not shown

^2^ Hydrogen bonds found in the X-ray structure (PDB code 1T6O)

To further investigate the importance of the effect of the substitutions on the binding affinity, additional MD simulations were carried out using the free energy perturbation (FEP) method (see details of the method in the Materials and Methods section). The calculations were based on the thermodynamic cycle shown in **S3 Fig** which allowed us to estimate the impact of an amino acid substitution on the binding energy by measuring the ΔΔG between the *wt* and mutated complexes at 300K. Replacement of Ser491 of N_TAIL_ by Leu led to an average binding free energy change ranging from 3.22 to 3.91 kcal.mol^-1^. These ΔΔG values correspond to a 200-fold to 700-fold reduction in binding affinities for the S491L variant which is compatible, although a bit more pronounced, with the K_D_ calculated for this variant using the empirically determined equation between luminescence and K_D_ values (see above and **Fig 4**). In a similar manner, substitution of R497 with Gly led to ΔΔG values ranging from 1.29 to 1.85 kcal.mol^-1^. This corresponds to a 10 to 20-fold reduction of binding affinity which nicely correlates with the K_D_ reduction-fold as measured by ITC.

**Fig 5:**
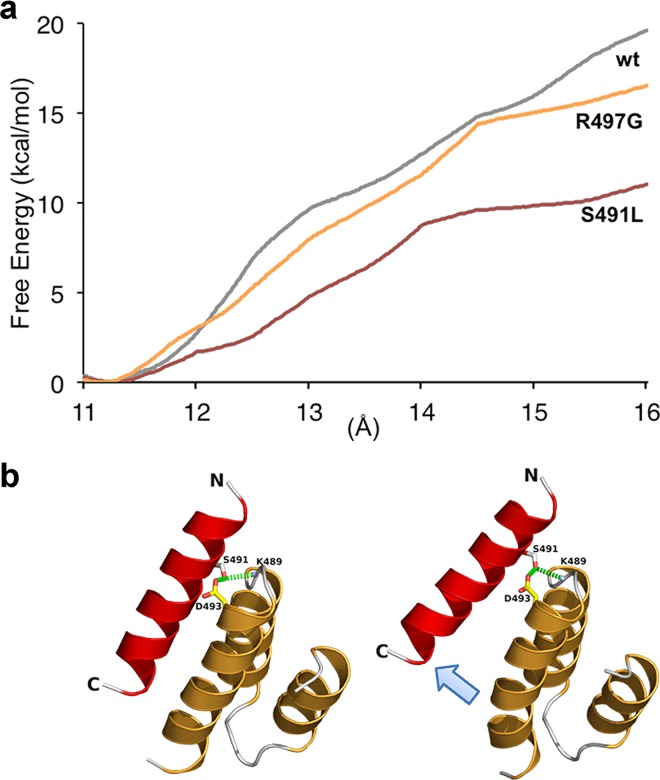
Dissociation of wt XD/α-MoRE complex computed with the adaptive bias force (ABF) molecular dynamics method. **(a)** Free energy profile for wt complex and the two R497G and S491L variants. **(b)** Snapshots of the wt complex illustrating the dissociation through the C-terminal end of the α-MoRE (light blue arrow). XD and N_TAIL_ are represented in cartoon mode and colored orange and red, respectively. Hydrogen bonds involving S491 of N_TAIL_ are shown as green dashed lines. See also **S2 and S3 Figs**.

The dissociation of the XD/N_TAIL_ complex cannot be observed during the time course of free MD simulations. To obtain more insights into the dissociation process, we therefore performed simulations using adaptive biasing force (ABF), a method that allows overcoming barriers of the free-energy landscape [57]. The center of geometry between the two partners was selected as ordering parameter and both proteins were allowed to diffuse reversibly along this reaction coordinate during the different stages of the simulations (no average force was exerted along the ordering parameter). The free energy profiles of the *wt* and mutated complexes are shown **Fig 5A**. The global minimum corresponds to a distance around 11.3 Å, very close to the distance observed in the X-ray structure (11.03 Å). Analysis of the *wt* complex reveals that the dissociation between the two partners proceeds from the C-terminal part of N_TAIL_ corresponding to the more hydrophobic residues (**Fig 5B** and **S2 Fig**). The final step of the dissociation corresponds to the disruption of hydrogen bonds between Ser491 of N_TAIL_ and Lys489 and Asp493 of XD. The R497G complex exhibits an energy profile similar to that of the *wt* complex with slightly lower energy values indicating a lower resistance against disruption. In the case of the S49IL complex, the disruption can occur from either end of the α-helix of N_TAIL_ depending on the trajectory. This behavior can be explained by the loss of hydrogen bonding with Lys489 and Asp493 of XD. As a consequence, the energy profile is profoundly affected and this variant shows less resistance toward disruption.

Altogether, these data provide a mechanistic basis illuminating the critical role played by N_TAIL_ residues Ser491 and Arg497 in stabilizing the N_TAIL_-XD complex.

### Ability of N variants to support re-initiation of transcription at intergenic regions as a function of N_TAIL_/XD binding strength

**Fig 6:**
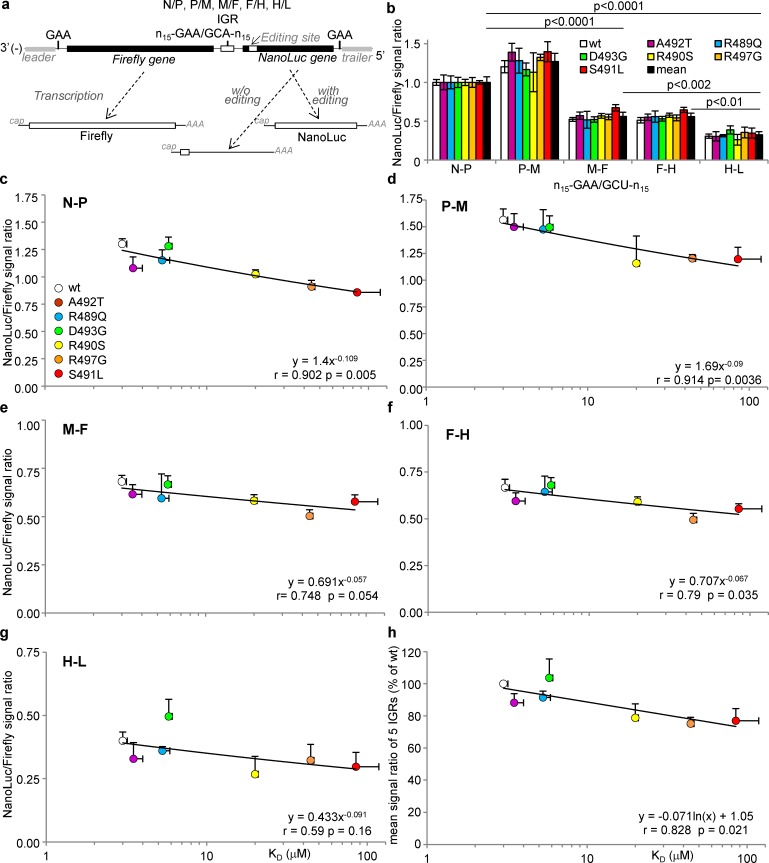
Ability of N variants to support transcription re-initiation at every MeV intergenic region (IGR) as determined using dual-luciferase 2-gene minigenomes coding for Firefly and NanoLuc luciferases. (**a**) Schematic structure of the minigenomes encoding the Firefly luciferase gene at the 3’ end of the genomic sequence just downstream the leader and N gene UTR as a first gene and NanoLuc luciferase as a second reporter gene. NanoLuc luciferase is conditionally expressed by MeV polymerase mediated-edition of the transcript thanks to an editing site grafted just after the AUG codon in such a way that, without the non-templated addition of one G, the downstream coding sequence is out of frame because of the presence of an in-frame stop codon. The two genes are separated by either the N 5’UTR and the P 3’UTR separated by the natural N-P un-transcribed 3’-GAA-5’ triplet that characterizes the N-P IGR region or by P-M, M-F, F-H or H-L IGR regions, i.e. un-transcribed 3’-GAA-5’ (or 3’-GCA-5’ for H-L) triplet flanked by canonical upstream and downstream gene end and gene start sequences arbitrarily fixed to 15 nt. (**b**) Homogenous and comparable decrease of the efficiency of the transcription re-initiation mediated by every MeV IGR from 3’ to 5’ gene position observed with every N variant when the NanoLuc/Firefly signal ratio is normalized as a function of that observed with the first N-P IGR. MeV IGRs can be grouped into three subsets of re-initiation efficiency, high for N-P and P-M, medium for M-F and F-H and low for H-L. (**c**-**g**) Variation in the efficiency of re-initiation as a function of the 5 different MeV IGRs as determined from the NanoLuc/Firefly signal ratios (i.e. same data as in (**b**) but without normalization to the N-P IGR) and expressed as a function of K_D_ of the N_TAIL_/XD pair. Note the progressive loss of correlation from N-P to H-L IGRs. (**h**) Correlation of the mean re-initiation rate through the five MeV IGRs (estimated as mean NanoLuc/Firefly signal ratio observed over the five IGR regions) with N_TAIL_/XD K_D_. Minigenome data are expressed as the mean +/- SD of at least 3 independent experiments, with each combination being done in triplicate. See also **S5 Fig**.

In order to investigate the functional consequences of attenuating the interaction between N_TAIL_ and XD we tested the ability of each N variant to support the expression of a reporter gene from a minigenome rescued into a functional nucleocapsid by cotransfecting a plasmid coding for the minigenome under the *T7* promoter together with P and L expression plasmid [58]. To take into account the transcription re-initiation at IGRs, we conceived and built new dual-luciferase minigenomes coding for Firefly and *Oplophorus gracilirostris* (NanoLuc) luciferase as first and second reporter gene respectively separated by each of the five IGRs of MeV genome (**Fig 6A**). To this end, the NanoLuc luciferase was chosen because it has a ~150-times higher specific activity compared to Firefly luciferase [59]. Like many other paramyxoviruses, MeV polymerase has the ability to edit P mRNA by adding one non-templated G when transcribing the specific sequence termed P editing site (3’-uguggguaauuuuuccc-5’) [12,60]. We introduced this editing site just downstream the 3’-UAC-5’ START codon of the NanoLuc gene so as to condition the creation of the NanoLuc ORF and the ensuing translation of NanoLuc to the co-transcriptional insertion of one non-templated G by MeV polymerase. If minigenome RNA transcripts made by the T7 RNA polymerase are basally translated in spite of the lack of both cap and polyA signals (**S4A Fig**), the T7 RNA polymerase does not recognize the P editing signal [61]. As a result, while the signal to noise ratio is ~24 for Firefly, it reaches ~521 for the edited NanoLuc, i.e. a 20-fold increase of the dynamic range (**S4B Fig**).

As a measure of the efficacy of each N variant to support the rescue of each minigenome, Firefly luciferase signals specifically driven by MeV polymerase from the first gene (as obtained after subtraction of background levels observed in the absence of a functional L, (see **S5 Fig**)) were compared. They were all found to be of similar magnitude irrespective of the MeV IGR within the minigenome and of the N variant, thus indicating comparable efficiencies of the rescuing step which relies on the random but ordinated encapsidation by the N protein of the naked RNA minigenome transcribed by the T7 polymerase ([62] see [63] for review) (**S5A and S5B Fig)**.

We then verified that these newly built dual-luciferase minigenomes harboring individually one of the five IGR faithfully reproduce the expected re-initiation strength gradient. Indeed, when normalized to the NanoLuc/Firefly signal ratio observed with a minigenome carrying the N-P IGR, the ratios observed for the minigenomes harboring the downstream IGRs decrease with their remoteness from the genome 3’end with P-M being equivalent to N-P, M-F and F-H being significantly lower and H-L being the lowest of all (**Fig 6B**, *wt* **N**). These results are in agreement with the transcription gradient observed in MeV infected cells [12,64,65,66] and with the efficacy of Sendai virus re-initiation at each IGR as determined using recombinant viruses [67]. Interestingly, this trend was absolutely conserved for every N_TAIL_ variant upon normalization to the ratio observed with N-P IGR minigenome (**Fig 6B**) indicating that the observed re-initiation strength gradient is an intrinsic property of each IGR region. When NanoLuc/Firefly ratios observed for each N variant were plotted without normalization as a function of N_TAIL_/XD binding strength for each of the five MeV IGR minigenome, the NanoLuc/Firefly signal ratio was found to decrease with decreasing binding strength, with the correlation being significant at p~0.05 or below for N-P, P-M, M-F and F-H IGR minigenomes (**Fig 6C–6E**), and the trend conserved for the minigenomes bearing the remotest H-L IGR (**Fig 6F and 6G**). Since in the natural situation MeV polymerase has to travel through every IGR, we estimated for each individual variant a mean re-initiation rate through all MeV IGRs by calculating the mean NanoLuc/Firefly ratio of the 5 IGR regions for each N variant. Remarkably this mean re-initiation rate correlates with the N_TAIL_/XD binding strength (**Fig 6H**, p = 0.021).

### Ability of N variants to misrecognize IGR and generate read-through transcripts

**Fig 7:**
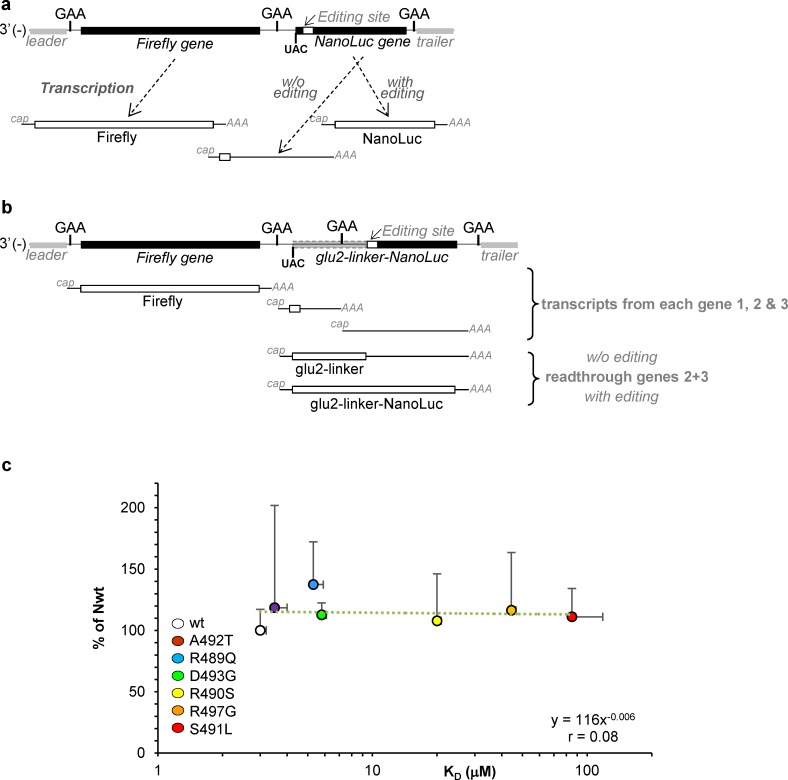
Ability of N variants to misrecognize IGR and generate read-through transcripts. Schematic structures of 2-gene **(a)** and 3-gene **(b)** N-P minigenomes. See Fig 6A for detailed description of 2-gene minigenome. The 3-gene minigenome comprises from its 3’-end the Firefly CDS as a first gene, a second gene encoding the C-terminal domain of Gaussia luciferase lacking a stop codon and a third gene encoding the NanoLuc CDS devoid of start codon and in frame with the upstream Gaussia CDS only after the upstream insertion of one additional G by RNA editing. Consequently the expression of the NanoLuc luciferase (as chimeric Glu2-linker-NanoLuc protein) requires the re-initiation of the transcription at the IGR separating gene 1 and gene 2, the reading through the IGR separating gene 2 and 3 and the edition of the read-through transcript. Therefore, if the expression of NanoLuc luciferase from both 2-gene and 3-gene minigenomes relies on both the accuracy of the re-initiation of the second gene over the same N-P IGR and the editing of the transcript, the expression of the NanoLuc luciferase from the 3-gene minigenome additionally relies on the efficiency of the read-through between gene 2 and gene 3. **(c)** Ratios of NanoLuc signals (normalized by their upstream Firefly signal) observed with each N variant with 3-gene (numerator) and 2-gene (denominator) minigenome observed with each N variants. Data are expressed in % of the ratio observed with wt N.

In few percent cases, the viral polymerase fails to recognize an intergenic region. This results in read-through transcripts. To investigate the possible impact of N_TAIL_/XD binding on read-through generation, a 3-gene minigenome was built as follows: the first gene code for the Firefly luciferase, the second gene codes for an irrelevant inactive protein (here the C-terminal half of the Gaussia luciferase (Glu2)) followed by a linker that remains in the same coding phase throughout the second downstream N-P IGR and the third gene which contains the NanoLuc luciferase coding sequence devoid of a start codon and out of frame by one missing nucleotide that can be restored by the editing signal. Consequently, among all possible viral transcripts, only the edited read-through mRNA over gene 2 and gene 3 can give rise to a NanoLuc luciferase activity (**Fig 7A and 7B**). Therefore, with the 3-gene minigenome, the NanoLuc/Firefly ratio is dependent on two IGR-related effects: the re-initiation of the transcription at the first IGR and the failure to recognize the second. As expected, the NanoLuc/Firefly signal ratios obtained with this 3-gene minigenome were found to be of a much lower level (i.e. few percent) than those observed with the 2-gene minigenome shown in **Fig 6C**. We normalized the NanoLuc/Firefly signal obtained with the 3-gene minigenome by the signal obtained with the 2-gene minigenome in order to cancel out the effect on the re-initiation at the first IGR and to focus on the generation of read-through transcripts at the second IGR. The resulting ratios are similar for all the variants, thus indicating they all roughly produce the same amount of read-though transcripts (**Fig 7C**). We conclude that the N_TAIL_/XD binding strength does not significantly impact the failure of the viral polymerase to recognize the N-P intergenic region.

### Ability of N variants to support re-initiation of transcription during polymerase scanning over an elongated un-transcribed IGR

**Fig 8:**
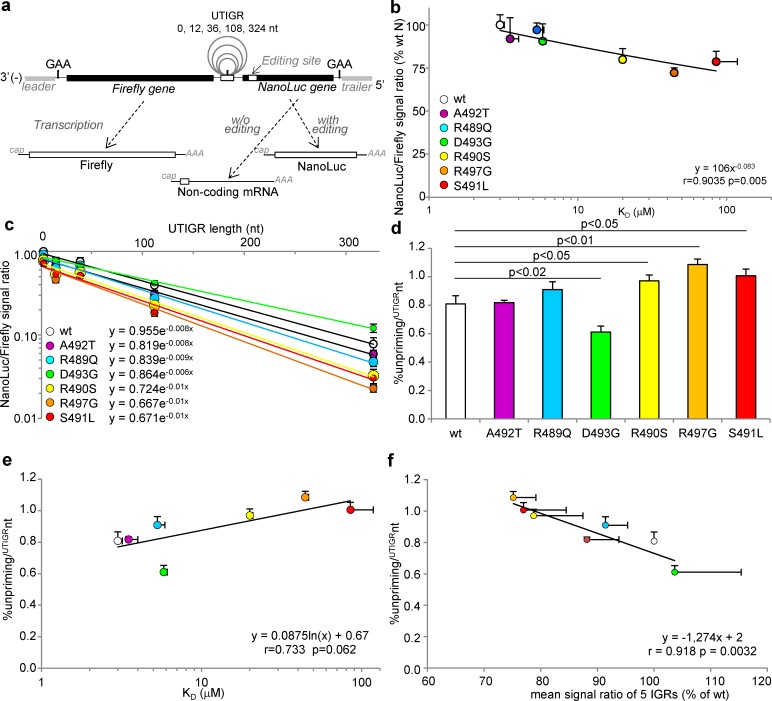
Ability of N variants to support transcription re-initiation over untranscribed IGRs (UTIGR) of variable length as determined using dual-luciferase minigenomes. **(a)** Principle of the dual-luciferase editing-dependent minigenome assay allowing the expression of the Firefly as the first gene and the NanoLuc as the second gene, the translation of which relies on the addition of one non-templated G at the editing site (see **Fig 6A** for details). The two genes are separated by a modified N-P IGR where the polyadenylation site is ended by a canonical G and is followed by 12, 36, 108 or 324 nucleotides followed in their turn by a modified inactive polyadenylation site and the canonical 3’-GAA-5’ intergenic triplet i.e. 3’-auauuuuuuG[n]auCauuuuuuGAA-5’ as validated for SeV minigenomes [70] (**b**) Correlation between NanoLuc/Firefly signal ratios and N_TAIL_/XD K_D_ as observed with N-P intergenic minigenome (i.e. UTIGR n = 0). **(c)** NanoLuc/Firefly signal ratio observed with individual N_TAIL_ variants as a function of UTIGR length. The correlation is statistically significant at p<0.001 for all N variants. (**d**) Calculated unpriming rate per un-transcribed intergenic nucleotide from data shown in **(c)** and **(e)** their relationship with the N_TAIL_ to XD binding strength. **(f)** Correlation between unpriming rate per un-transcribed intergenic nucleotide and mean re-initiation rate through the five MeV IGR regions (estimated as mean NanoLuc/Firefly signal ratio observed over the five IGR regions, as also shown in **Fig 6H**). Minigenome data are mean values and SD from three independent experiments, each data point being made in triplicates. Statistically significant differences are as determined using the Student’s t or Spearman’ R test. See also **S7 Fig**.

Upon crossing an IGR, the polymerase from *Mononegavirales* having ceased RNA synthesis at the GE is able to scan forward and backward the genome template until it recognizes the transcription re-initiation site GS of the next downstream gene. This search for next GS had been initially observed as measurable temporal pause in transcription [68] (see viral transcription scheme in **S6 Fig** and [13,69] for reviews). Since the frequency of re-initiation decreases with the length of the un-transcribed IGR (UTIGR) [70,71] dual-luciferase Firefly/NanoLuc 2-gene minigenomes with elongated UTIGR based on MeV N-P IGR were also built (see scheme **Fig 8A**) according to previous work based on the related Sendai virus that has served as the reference study model for *Paramyxoviridae [70]*. The Firefly signals specifically driven by MeV polymerase (as obtained after subtraction of background levels observed in the presence of an inactive L protein) observed with each combination of minigenome of variable UTIGR length and N variant were of similar magnitude irrespective of the UTIGR length and of N_TAIL_ variant (**S7A Fig)** and did not show any correlation with the N_TAIL_/XD binding strength (**S7B Fig)**. These data confirmed that the rescue of the minigenome, is neither dependent on the sequence of the minigenome nor on the N variant. Incidentally, these experiments also allowed appreciating the reproducibility of our dual-luciferase minigenome-based experiments, as judged by comparing **S5A and S7A Figs**.

As observed in the previous set of experiments, the NanoLuc/Firefly signal ratios obtained with the N-P minigenome (i.e. UTIGR “+0”) nicely correlate with the N_TAIL_/XD binding strengths (**Fig 8B**, compare also with **Fig 6C** for data reproducibility). When N variants were tested with elongated UTIGR minigenomes, the NanoLuc/Firefly signal ratio exponentially declined with UTIGR elongation (**Fig 8C,** p<0.001 for every N variant). However the declining rate varied between N variants (compare the slopes in **Fig 8C**). This allowed us to calculate and compare the percentage of unpriming *per* UTIGR nt (%unpriming/^UTIGR^nt). The D493G variant exhibits a significantly lower %unpriming/^UTIGR^nt compared to *wt* N, whereas that of R490S, R497G and S491L variant was significantly higher (**Fig 8D**). Furthermore, the %unpriming/^UTIGR^nt of N variants tends to vary according to the *log* of the N_TAIL_/XD K_D_, (**Fig 8E**, p = 0.062). Remarkably, the %unpriming/^UTIGR^nt and mean re-initiation rate through the five MeV IGR regions significantly correlate to each other (**Fig 8F**, p = 0.0032). Overall these data reveal that lowering the N_TAIL_/XD binding strength significantly increases the unpriming rate of MeV polymerase during transcription re-initiation and its scanning over un-transcribed genomic sequences, i.e. over each UTIGR.

### Impact of N_TAIL_ amino acid substitutions introduced into recombinant unigene and biG-biS viruses on virus production

**Fig 9:**
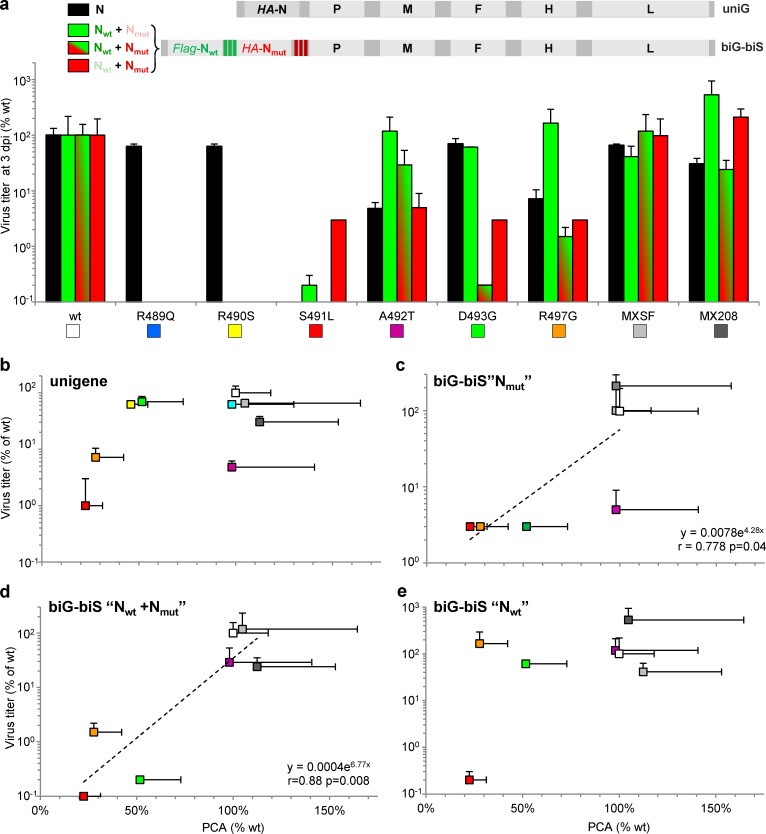
Virus production at 3 d.p.i. and in the context of unigene and biG-biS viruses and relationships with N_TAIL_/XD interaction strength. **(a)** Infectious virus production after infection with recombinant virus coding for N_TAIL_ variant (unigene virus) or biG-biS virus bearing two copies of the N gene, one coding for wt Flag-N_1_ and either wt or variant HA-N_2_ in conditions allowing selective expression of the wt Flag-N_1_ copy, wt or variant HA-N_2_ copy or both of them (data expressed in % of wt, mean ± SD). Although all biG-biS viruses were successfully rescued, R489Q, R490S and S491L could not be further studied because of a too low virus production. **(b-e)** Virus titers expressed in % of wt virus (for unigene viruses) or wt Flag-N_1_/wt HA-N_2_ (for biG-biS viruses) were plotted (mean ± SD) against N_TAIL_/XD binding strength as determined by glu-PCA (mean ± SD). Panel **b** shows virus production from unigene N variants. Virus production from biG-biS viruses with **(c)** selective expression of variant HA-N_2_ copy, or **(d)** simultaneous expression of wt Flag-N_1_ and variant HA-N_2_ copies, or **(e)** selective expression of the wt Flag-N_1_ copy. Same color codes as in **Fig 1B**, see also panel a. Shown are means and SD as obtained from three independent infections. See also **S8, S9 and S10 Figs**.

Since even N_TAIL_ variants with the highest K_D_ for XD were able to reconstitute functional dual-luciferase minigenomes, we sought at evaluating the impact of substitutions in the viral context by expressing N variants into two types of recombinant viruses, namely unigene and biG-biS viruses. Unigene viruses possess only one copy of the N gene and thus express solely the mutated N protein. By contrast, biG-biS viruses contain a duplicated viral gene, here the N gene, one encoding the *wt* N protein (*wt* Flag-N1) and one encoding the mutated N protein with a HA tag (HA-N2), the expression of which can be independently silenced thanks to the use of two cell lines expressing shRNA that selectively target one of the two N genes (**S8 Fig)** [48]. Unigene viruses harboring N_TAIL_ variants were all rescued. The biG-biS viruses were also all rescued in cells allowing the selective expression of the *wt* Flag-N1 gene copy, although the too low virus production by the R489Q and R490S viruses prevented further analysis. Virus production by recombinant viruses at 3 d.p.i. were determined for unigene viruses in Vero cells, while that of biG-biS viruses was measured in three host cells allowing selective expression of either the *wt* Flag-N1 gene copy, the HA-N2 gene variant, or both of the N gene copies simultaneously (**Fig 9A**). Virus production was found to be very low (at least 2 log reduction with respect to the *wt* counterpart) in the case of unigene and biG-biS S491L viruses. Note that the possibility that the observed differences in virus production of unigene viruses could be ascribed to a defect in N variant expression (**S1 and S9A Figs)** or to a significant contamination by defective interfering (DI) mini-replicons was checked (**S9B–S9D Fig)** and ruled out.

When plotted against the N_TAIL_/XD binding strength as determined by glu-PCA, the virus production of unigene N_TAIL_ variants does not significantly correlate with binding strength (**Fig 9B**). However, the virus titer of biG-biS viruses under the selective expression of HA-N2 variant and under the combined expression of both N copies were found to correlate with N_TAIL_/XD binding strength (p = 0.04 and p = 0.008, respectively) (**Fig 9C and 9D**), while no such a correlation was found upon selective expression of the *wt* Flag-N1 copy as expected (**Fig 9E**). We noticed that the coexpression of N *wt* with D493G variant appears deleterious for virus production (**Fig 9D**). However, in a minigenome assay such a mixture of N was as efficient as N *wt* alone (**S10 Fig)**, thus ruling out the possibility that N_TAIL_ heterogeneity could directly impact the polymerase activity. Overall these data indicate that the N_TAIL_/XD binding strength may control the virus production to some extent.

### Viral transcription gradient correlates with XD-binding affinity of Box2 variants

**Fig 10:**
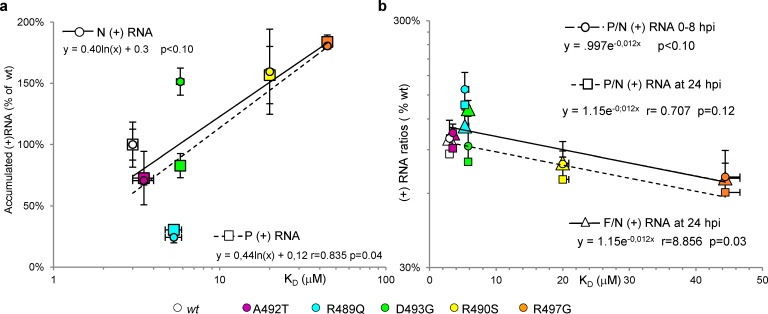
Relationship of transcripts accumulation rates and transcripts ratios with N_TAIL_/XD binding affinity. Relationship of **(a)** accumulation rate of N (+) or P (+) RNA during primary transcription with N_TAIL_/XD binding affinity and of **(b)** P/N or F/N (+) RNA ratios at either 0–8 h.p.i. or at 24 h.p.i. with N_TAIL_/XD binding affinity. Data are expressed as % of wt. See also **S11 Fig.**

We then took advantage of unigene viruses expressing the single-site Box2 variants to determine which activity of the viral polymerase could be affected by a change in the N_TAIL_/XD binding affinity. Vero cells were infected with *wt*, R489G, R490S, A492T, D493G and R497G unigene viruses. Note that the S491L variant was not investigated since it could not be further amplified to reach a workable titer. RNA synthesis parameters reflecting primary transcription (i.e. mostly, if not solely, transcription, mediated by the active polymerases brought by infecting virions), secondary transcription and replication were determined by quantification of (+) and (-) RNA accumulation at different times post-infection as previously reported [48,65]. When RNA synthesis parameters were plotted along with N_TAIL_/XD K_D_, it appeared that both (+) RNA transcript accumulation rate and ratios between P (or F) and N transcripts could be roughly predicted from the interaction strength between the N_TAIL_ variant and XD as measured by either method (**Fig 10**). The correlations were statistically significant between the accumulation rate of P (+) transcripts and N_TAIL_/XD K_D_ (**Fig 10A**) and between the F/N transcript ratios measured at 24 h.p.i. and the K_D_ (**Fig 10B**). In further support of the coherence of the results, a good correlation was found between the accumulated levels of N and P (+) RNAs during primary transcription and at 24 h.p.i. (**S11A and S11B Fig**), and between both N (+) and P (+) RNA transcripts and (-) genomic RNA (**S11C and S11D Fig**).

**Fig 11:**
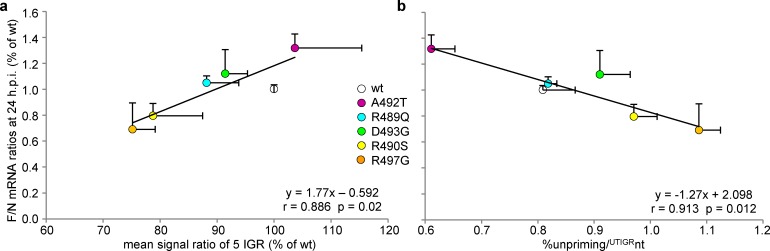
**Relationship of F/N mRNA ratio at 24 h.p.i. from unigene viruses with the mean re-initiation rate over the five IGRs (a) and the %unpriming/** ^**UTIGR**^ **nt (b)** (from data shown in **Figs 6, 7 and 9**).

When the F/N mRNA ratios at 24 h.p.i. observed with unigene viruses were plotted against the calculated mean re-initiation rate of the 5 IGRs and the %unpriming/^UTIGR^nt a significant positive and a negative correlation were found, respectively (**Fig 11)**. Altogether, these data support that the N_TAIL_/XD binding strength controls, at least in part, the steepness of the viral transcription gradient.

## Discussion

By combining *in vitro* biophysical and biochemical studies, *in silico* analyses (i.e. MD simulations) and *in cellula* polymerase functional investigations using recombinant viruses and dual-luciferase editing-dependent minigenome assays, we deciphered key molecular parameters that govern the N_TAIL_/XD interaction. Specifically, we uncovered a correlation between interaction strength and efficiency of transcription re-initiation at intergenic regions.

### Impact of preconfiguration of the α-MoRE on the interaction with XD

For most of the N_TAIL_ variants the observed variations in binding affinities cannot be ascribed to differences in the extent of α-helical sampling of the free form of the α-MoRE, nor to differences in the ability of the latter to undergo induced α-helical folding. However, the R489Q substitution represents an exception in this respect: indeed, it has a reduced extent of α-helicity and a slightly increased K_D_ towards XD. The reduced α-helical content of this variant is in line with secondary structure predictions, as obtained using the Psipred server (http://bioinf.cs.ucl.ac.uk/psipred/) [72], that predicts a slightly lower helical propensity. Whether the experimentally observed reduction in affinity towards XD arises from this lower helicity or from other attributes, including charge-related ones, remains to be established. This variant also displays a reduced accumulation rate of primary transcripts. The subtle molecular mechanisms underlying the peculiar behavior of this variant remain however to be elucidated.

### N_TAIL_ binding to XD involves a hydrogen bonding network including three α-MoRE and three XD residues

The complex hydrogen bonding revealed by MD simulations of N_TAIL_/XD complexes allows the drops in binding affinities experimentally observed for the S491L, R497G and R490S variants to be rationalized. Interestingly, these substitutions, which have the most dramatic effects in terms of binding affinities, are also the ones that have the strongest effect on virus replication, with the S491L substitution being very poorly tolerated even in biG-biS viruses. The poor ability of the low-affinity S491L variant in mediating efficient virus replication is reminiscent of the comparable deleterious effect of the F497D XD substitution [48] and of the detrimental effect of the deletion of the N_TAIL_ region encompassing the α-MoRE [49].

### The lower the N_TAIL_/XD binding strength the lower the efficiency of transcription re-initiation at intergenic regions

We provide here compelling evidence indicating that the strength of the N_TAIL_/XD interaction controls, at least in part, the ability of the P+L polymerase complex to re-initiate at IGRs: data obtained using our highly sensitive and reproducible dual-luciferase minigenome assay reveal a significant correlation between the N_TAIL_/XD binding strength and the efficiency of the transcription re-initiation. Since our minigenome assays rely on the edition of the second reporter gene, we cannot formally exclude that the editing may be also impacted by the N_TAIL_/XD binding strength. However, the calculated %unpriming/^UTIGR^nt only depends on the decrease of the NanoLuc/Firefly signals ratios with the length of the UTIGR. The observed effect is therefore independent of any potential effect on the edition (i.e. if N mutations only had an effect on editing, then this effect should be the same irrespective of the IGR under study and of its length, which is not the phenotype we observed). Moreover, the correlation in the viral context between the P/N and F/N mRNA ratio and the K_D_, supports a role for the XD/NTAIL interaction strength in the re-initiation at IGRs.

A N protein truncated of its last 86 C-terminal amino acids, i.e. truncated of most of N_TAIL_ including the XD binding site, had been shown to be active in transcription and replication both in a minigenome assay and when introduced into a recombinant virus [49]. We confirmed that the N1-439 truncated protein is as good as, if not better than, the *wt* N in transcribing the Firefly gene from our N-P 2-gene minigenome construct (**S12A Fig**). However, its ability to support transcription re-initiation over the N-P junction was significantly reduced, with the extent of reduction being comparable with that observed with the low affinity R497G variant (**S12B Fig**, UTIGR 0 nt), thus confirming the role of N_TAIL_/XD interaction in transcription re-initiation. This low efficiency of transcription re-initiation may explain the extreme growth defect of the recombinant virus bearing the truncated N until reversion to a *wt* N [49].

**Fig 12:**
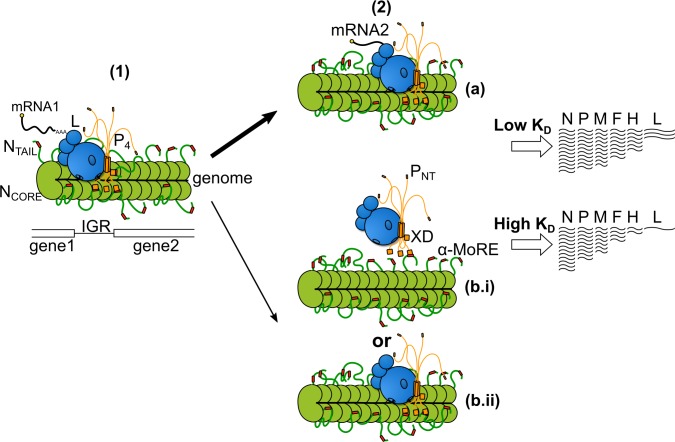
Model of transcription re-initiation. (**1**) The polymerase complex, composed of L and P proteins, transcribes the genome. (**2**) After addition of the poly(A) tail and release of the mRNA, the polymerase complex may re-initiate transcription and transcribe the next gene **(a)** or stop transcribing **(b).** Whether the polymerase complex detaches from the genome template **(b.i)** or travels on it until reaching the 5’ end of the genome **(b.ii)** remains to be determined. The higher is the K_D_, the less efficient is the re-initiation of transcription, thus leading to a steeper mRNA gradient.

Assuming a very slow degradation of viral mRNA [65,66,73], the transcripts accumulation rate in cells infected with unigene viruses reflects the RNA synthesis rate by the polymerase, the number of active polymerases (and their recruitment onto the nucleocapsid template), and the number of polymerases that are recruited per time unit on a given gene. For the same reason, the transcript ratios between the different genes are likely mostly governed by the efficiency with which the polymerase re-initiates the transcription at each IGR. Assuming this being a conserved feature for every N variant, we can reasonably interpret the inverse correlation we observed between multiple transcript ratios and K_D_ as reflecting a direct control of the N_TAIL_/XD binding strength on the efficiency of the re-initiation at each IGR. A lower binding strength leads to lower levels of downstream transcripts. After completion of the polyadenylation of the messenger encoded by the upstream gene, the polymerase may remain firmly in contact with its genomic RNA template embedded into the nucleocapsid only if maintained by the anchoring of its P subunit via a dynamic binding of its X domain to the TAIL domain of N subunits located at the IGR (**Fig 12**). Therefore, a decrease in the XD/N_TAIL_ affinity may favour the unpriming of the polymerase. Whether unprimed polymerases can detach from the nucleocapsid or stay on the template and move forward to the end of the nucleocapsid remain to be established. Hence, XD to N_TAIL_ anchoring would tightly control the re-initiation level of the RNA synthesis by the polymerase in the transcription mode, thus determining the steepness of the transcription gradient (**Fig 12,** see also **S6B Fig**).

### The N_TAIL_/XD affinity affects the processivity of the polymerase both on the “transcription” and on the “scanning” modes

What could be the functional significance of the relationship between the accumulation rate of primary N and P transcripts and the XD/N_TAIL_ binding strength? As speculated, the dynamics of XD/N_TAIL_ binding and release may also affect the polymerase processivity on the nucleocapsid [48]. The XD/N_TAIL_ interaction may act as a brake and slows down the polymerase: the weaker is the interaction, the weaker is the brake. Also, because of the efficient recycling of the polymerases on the promoter [65], if, in the absence of transcription re-initiation, the polymerase detaches from the RNA template, a steeper gradient would release more polymerases available for transcription of the first genes. With weaker N_TAIL_/XD interactions, the viral production by unigene viruses tends to be negatively affected although the correlation was not statistically significant likely because of the small number of available virus variants and the too high variability of the result due to the multiple intervening parameters (see **S6A Fig** and the complete scheme of virus replication dynamics in [65]). However with biG-biS viruses, we did observe a significant correlation between virus production and N_TAIL_/XD binding strength in conditions where the N variant was selectively expressed. This significance may reflect both the higher number of available virus variants and/or the higher impact of the modulation of the transcription re-initiation process in viruses possessing an additional transcription unit (i.e. where the polymerase has to go through one additional IGR). The similar correlation observed upon the co-expression of both *wt* Flag-N1 and variant HA-N2 copies may indicate similar impact on transcription re-initiation because of the tetrameric valence of the P anchoring on (contiguous?) heterogeneous N_TAIL_ appendages. Alternatively, it is possible that the heterogeneity of N_TAIL_ within a given nucleocapsid template may have a negative impact on other mechanisms such as nucleocapsids packaging into particles since N_TAIL_ also recruits the M protein [74], a key virion assembly factor [75]. The discrepancy we observed between virus production from biG-biS viruses and minigenome data with mixed N_TAILs_ argues for this later hypothesis.

Using minigenomes with elongated UTIGR, we were able to measure the unpriming rate of the polymerase in the “scanning mode” and we show that a decrease in the N_TAIL_/XD affinity induces an increase of the unpriming rate. In this situation, without the stabilization and the active motion of the polymerase due to the RNA synthesis, the role of the N_TAIL_/XD interaction in maintaining the polymerase on the nucleocapsid may overcome the “brake” effect. Alternatively, as suggested by Krumm *et al* [49], the N_TAIL_ may need to be rearranged by P to allow an efficient RNA synthesis. In this case, a too low N_TAIL_/XD affinity may weaken the efficiency of P in rearranging N_TAIL_ and would favor the unpriming of the polymerases. The fact that the N1-439 variant, that lacks most of N_TAIL_, has the lowest unpriming rate on UTIGR supports this second hypothesis (0.6 vs 0.81%unpriming/^UTIGR^nt for N1-439 and *wt* N respectively) (**S12C Fig)**.

### A mechanism among others to control both scanning and re-initiation by MeV polymerase

In conclusion, the XD/N_TAIL_ interaction may play a critical role in the polymerase processivity, in maintaining the polymerase anchored to the nucleocapsid during its scanning upon crossing the intergenic regions, and/or in the transcription re-initiation at each intergenic region. Since both increasing [48] or decreasing (this study) the XD/N_TAIL_ affinity negatively affect the viral growth, the wild type XD/N_TAIL_ binding strength seems to have been selected to mediate an optimal equilibrium between polymerase recruitment, polymerase processivity and transcription re-initiation efficiency. A corollary of this is that substitutions that strongly affect affinity towards XD are poorly tolerated. Consistent with this, the substitutions with the most dramatic impact herein investigated (i.e. R490S, S491L and R497G) do not naturally occur in any of the 1,218 non-redundant MeV sequences, while those that have a less drastic impact (i.e. R489Q, A492T and D493G) are found in circulating measles strains [47]. Interestingly, in the case of Ebola virus (EBOV), an additional protein, i.e. VP30, serves as an anti-terminator transcription factor, and mutations that either decrease or increase the binding affinity between N and VP30, decrease RNA synthesis [76] thus arguing for a similarly tightly regulated interaction. According to our work, the N_TAIL_ to XD binding strength tightly controls the transcription gradient. However, this does not rule out the possibility that other mechanisms may be at work in controlling the steepness of the gradient. Indeed, in the brain of three patients suffering from subacute sclerosis encephalitis (SSPE) or measles inclusion bodies encephalitis (MIBE) the transcription gradient was found to be steeper than the one measured in *in vitro* infected cells [77] although the amino acid sequences of N_TAIL_ and XD were found to be unvaried [78]. Furthermore, in the absence of the C protein, a steeper transcription gradient is also observed [79]. These two lines of evidence advocate for a multi-parametric control of the transcription gradient.

### The conserved bipartite P to N interaction of *Paramyxoviridae* members is also shared by other families of the *Mononegavirales* order

The major role of the N binding site on the C-terminus of P has been postulated to mediate L anchoring to the nucleocapsid without understanding the implication of such anchoring on the polymerase and/or on the nucleocapsid dynamics. The need for an optimized interaction between the P and N proteins might be one of the major evolution constraints to which the polymerase machinery of MeV, and possibly of paramyxoviruses in general, is subjected. Our findings raise also the question as to whether binding of the C-terminus of P to the globular moiety of N, as observed in other *Mononegavirales* members, needs to be similarly controlled reflecting a similar functional role.

The bipartite nature of P to N binding (see scheme **Fig 1A**) is remarkably conserved throughout the *Mononegavirales* order [80]. An α-MoRE located at N-terminus of P binds to the C-terminal globular domain of the N_CORE_ to form the so-called N^0^P complex that is used by the polymerase as the encapsidation substrate. Solved N^0^P structures from members of the *Rhabdoviridae* family (vesicular stomatitis virus, VSV) [8], *Filoviridae* family (VP35, of EBOV) [81,82], *Pneumoviridae* family (human metapneumovirus, HMPV) [9] and *Paramyxoviridae* family (Nipah virus, NiV a *Henipavirus* member) [7], MeV a *Morbillivirus* member [5], mumps virus, MuV a *Rubulavirus* member [83] revealed a common mechanism whereby the N terminus of P competes out with N arms that stabilize the oligomeric form of N and directly or indirectly prevents RNA binding. Structural and functional evidences indicate that, *via* its N-terminus, P can transiently uncover the genome at its 3’end from the first N subunits to give L access to its genomic RNA template (see [83] and [84] for review).

An additional N-binding site is located at the C-terminus of P (or VP35 for EBOV) (see scheme **Fig 1A**) [85] and allows binding to the assembled form of N. While this secondary binding site is required for the polymerase activity in minigenome experiments from several viruses [3,83,85,86,87], the structures of the reciprocal N-binding and P-binding site on P and N, respectively, look less conserved. In the case of NiV [42], Hendra virus [88], SeV [38,89] and MeV [27,30,44,89] the C-terminal domain of P (XD) is structurally conserved and consists of a bundle of 3 α-helices that are structurally analogous, and that dynamically binds to a α-MoRE located near the C-terminus of N_TAIL_ ([90,91], see [92] for reviews). This N_TAIL_-XD interaction is commonly characterized by a rather low affinity (K_D_ within the 3–50 μM range, [39,46,89] and this work). In *Rubulavirus* members, the C-terminal region of P spans in solution a structural continuum ranging from stable triple α-helical bundles to largely disordered, with crystal packing stabilizing the folded form [93,94]. In MuV, this triple α-helical bundle analogous to XD binds directly to the core of N subunits of the nucleocapsid [24] without excluding a complementary binding to the extremity of N_TAIL_ [83]. By analogy with MuV XD, MeV XD might also bind to another binding site located on N_CORE_. This would explain how transcription and replication can still be observed in the presence of the N1-439 truncated where interaction of P relies only on N_CORE_ ([49] and this paper). Indeed in other *Mononegavirales* members, the C-terminus of P binds to the core of N. In the case of RSV, the minimal nucleocapsid-binding region of P, which encompasses the last nine P residues, is disordered [95] and remains predominantly disordered even upon binding to the N-terminal lobe of N_CORE_ [96].

The C-terminal domain of P from Rabies virus (RABV) [97], Mokola virus [98,99] and VSV [100] share a fold made of a bundle of α-helices that binds to the core of two adjacent N proteins of the nucleocapsid [101,102]. The N protein of *Rhabdoviridae* members, along with the N protein from RSV lacks the disordered N_TAIL_ domain that characterizes N proteins from *Paramyxoviridae* members. In contrast to the XD-N_TAIL_ interaction, the C-terminal domain of RABV P binds to the nucleocapsid with a high affinity (K_D_ in the nanomolar range) [101]. In spite of the diversities of both structural features and binding modes within *Mononegavirales* members, does the binding of C-terminus of P to the assembled form of N fulfill common functions, namely ensuring the proper efficiency in polymerase scanning and re-initiation at intergenic regions? Further works will unveil to which extent our present findings are relevant for other members of the *Paramyxoviridae* or other families of the *Mononegavirales* order.

## Materials and Methods

### Plasmid construction

The pDEST17O/I vector [103], allowing the bacterial expression of N-terminally hexahistidine tagged recombinant proteins under the control of the T7 promoter, was used for the expression of all N_TAIL_ variants. The pDEST17 derivatives encoding single-site N_TAIL_ variants bearing substitutions within Box2 were obtained either by Gateway recombination cloning technology (variants R489Q, R490S, S491L and R497G) using the previously described pNGG derivatives [47] as the donor vectors, or by site-directed mutagenesis (variants A492T and D493G). In the latter case, we used a pair of complementary mutagenic primers (Operon) designed to introduce the desired mutation, Turbo-Pfu polymerase (Stratagene), and the pDEST17O/I construct encoding *wt* MeV (Edmonston B) N_TAIL_ as template [47]. After digestion with *Dpn*I to remove the methylated DNA template, CaCl_2_-competent *E*. *coli* TAM1 cells (Active Motif) were transformed with the amplified PCR product.

The pNGG derivative encoding the MXSF N_TAIL_ variant N-terminally fused to the N-terminal fragment of GFP was obtained in four steps using pNGG/N_TAIL_ as template [47,104] and site-directed mutagenesis PCR. In the first step, the pair of mutagenic primers was designed to introduce the first amino acid substitution. After PCR and *Dpn*I digestion, CaCl_2_-competent *E*. *coli* TAM1 cells (Active Motif) were transformed with the amplified PCR product. After having sequenced the construct to ensure that the desired mutation had been introduced, a second PCR was carried out using another pair of mutagenic primers designed to introduce the second substitution. Repeating this procedure four times led to the final construct bearing the four desired substitutions (i.e. D437V, R439S, P456S and P485L).

The sequences of the coding regions of all constructs generated in this study were checked by sequencing (GATC Biotech) and found to conform to expectations.

The pDEST17/N_TAIL_ construct encoding *wt* N_TAIL_ has already been described [47], as is the pDEST14 construct encoding C-terminally hexahistidine tagged MeV XD [30].

The plasmid p(+)MVNSe previously described in [48] was used as the MeV genome backbone. MeV genomic plasmids were built by direct recombination of one or two PCR fragments according to the InFusion user manual (Clontech). To build biG-biS recombinant viruses, the N gene was duplicated in N_1_ and N_2_ in gene positions 1 and 2, respectively. N_1_ was tagged with an N-terminal Flag peptide and three copies of the GFP RNAi target sequence (GAACGGCATCAAGGTGAA) in the 3’UTR of its mRNA. N_2_ was tagged with an N-terminal hemagglutinin (HA) peptide and three copies of the P RNAi target sequence (GGACACCTCTCAAGCATCAT) in the 3’UTR. Mutations into the N_TAIL_ domain of N, R489Q, R490S, S491L, A492T, D493G, R497G, MXSF (D437V/R439S/P456S/P485L) and MX208 (D437V/P485L/L524R), were introduced by subcloning PCR-amplified fragments from the pDEST17/N_TAIL_ vectors.

Full length *wt* and mutated N, *wt* and mutated N_TAIL_, P_PMD-XD_ and P_376-507_ fragments were subcloned downstream *Gaussia* glu1 and/or glu2 domains by InFusion recombination of PCR-amplified fragments as previously described [48]. All plasmids and viruses (N_1_, N_2_, P, M, and L gene) were verified by sequencing the subcloned PCR fragments or cDNA obtained by reverse transcription-PCR (RT-PCR) performed on virus stocks.

Plasmids encoding dual-luciferase editing-dependent 2-gene minigenomes were built by InFusion subcloning of PCR amplicons encompassing Firefly and NanoLuc coding sequences flanked by N UTR and L 3’UTR. The two luciferase coding sequences are separated by the N-P IGR either unmodified or exchanged with P-M, M-F, F-H and H-L IGRs (i.e. untranscribed 3’-GAA-5’ (or 3’-GCA-5’ for H-L) triplet flanked by canonical upstream and downstream gene end and gene start sequences arbitrarily fixed to 15 nt) or elongated by 12, 36, 108 or 324 nt (see sequences in **S2 and S3 Tables)** into the p107(+) MeV minigenome construct that drives the synthesis of (+) genomic strand under the control of the T7 promoter [62]. According to the rule of six that governs the strictly conserved hexameric length of measles virus genome [62,105], all minigenomes share identical phasing of the last U of the polyadenylation signal of the firefly gene (phase 6, i.e. the last nucleotide covered by the N subunit) and of the editing site with the C being in phase 6 as defined in [106]. A 3-gene minigenome coding for Firefly and chimeric Glu2-linker-NanoLuc luciferase as a results of read-through between gene 2 and gene 3 and RNA editing was built by modifying the N-P 2-gene minigenome. As a second gene, the ORF of the C-terminal domain of Glu (glu2) was inserted downstream to a START codon but without a STOP codon. This ORF is followed by a second N-P IGR and by the NanoLuc ORF without its own START codon, in frame “-1nt” to the upstream Glu2 ORF. Following addition of a G thanks to the presence of the P editing site, the NanoLuc ORF becomes in frame with the upstream Glu2 ORF. Consequently, the full-length chimeric Glu2-linker-NanoLuc can be uniquely translated from a read-through transcript over the second N-P IGR that is also edited (see sequence in **S4 Table**).

All plasmids will be deposited in the Addgene plasmid repository service except the glu1 and glu2 constructs that Addgene cannot accept. Those constructs are available upon request.

### Production and purification of N_TAIL_ and XD variants

The *E*. *coli* strain Rosetta [DE3] pLysS (Novagen) was used for the expression of all recombinant proteins. Transformants were selected on ampicillin and chloramphenicol plates. 50 mL of Luria-Bertani (LB) medium supplemented with 100 μg/mL ampicilin and 34 μg/mL chloramphenicol were seeded with the selected colonies, and grown overnight to saturation. An aliquot of the overnight culture was diluted 1/25 in LB medium containing ampicillin and chloramphenicol and grown at 37°C. When the optical density at 600 nm (OD_600_) reached 0.6–0.8, isopropyl ß-D-thiogalactopyranoside (IPTG) was added to a final concentration of 0.2 mM, and the cells were grown at 37°C for 4 additional hours. The induced cells were harvested, washed and collected by centrifugation (5,000 g, 12 min). The resulting pellets were frozen at –80°C.

All the N_TAIL_ and XD proteins were purified to homogeneity (> 95%) from the soluble fraction of bacterial lysates in two steps: Immobilized Metal Affinity Chromatography (IMAC), and size exclusion chromatography (SEC). Cellular pellets of bacteria transformed with the different expression plasmids were resuspended in 5 volumes (v/w) of buffer A (50 mM Tris/HCl pH 8, 300 mM NaCl, 20 mM imidazole, 1 mM phenyl-methyl-sulphonyl-fluoride (PMSF)) supplemented with lysozyme (0.1 mg/mL), DNAse I (10 μg/mL), 20 mM MgSO_4_ and protease inhibitor cocktail (Sigma). After a 30-min incubation with gentle agitation, the cells were disrupted by sonication. The lysate was clarified by centrifugation at 20,000 g for 30 min. The clarified supernatant, as obtained from a one-liter culture, was incubated for 1 h with 5 ml (50%) Chelating Sepharose Fast Flow Resin preloaded with Ni^2+^ ions (GE, Healthcare), previously equilibrated in buffer A. The resin was washed with buffer A supplemented with 1 M NaCl to remove contaminating DNA, and the proteins were eluted in buffer A containing 1 M NaCl and 250 mM imidazole. Eluents were analyzed by SDS-PAGE. Fractions containing the recombinant product were concentrated using centrifugal filtration (Centricon Plus-20, 5000 Da molecular cutoff, Millipore). The proteins were then loaded onto a Superdex 200 (N_TAIL_) or Superdex 75 (XD) 16/60 column (GE, Healthcare) and eluted in 10 mM Tris/HCl pH 8, 150 mM NaCl.

Protein concentrations were calculated using the theoretical absorption coefficients at 280 nm as obtained using the program ProtParam at the EXPASY server.

### Mass spectrometry

Mass analysis of the purified mutated N_TAIL_ proteins was performed using an Autoflex II ToF/ToF (Bruker Daltonics). Spectra were acquired in a linear mode. 15 pmol of samples were mixed with an equal volume (0.7 μL) of sinapinic acid matrix solution, spotted on the target and dried at room temperature.

The identity of the purified N_TAIL_ proteins was confirmed by mass spectral analysis of tryptic fragments obtained by digesting (0.25 μg trypsin) 1 μg of purified recombinant protein isolated onto SDS-PAGE. The tryptic peptides were analyzed as described above and peptide fingerprints were obtained and compared with *in-silico* protein digest (Biotools, Bruker Daltonics). The mass standards were either autolytic peptides or peptide standards (Bruker Daltonics).

### Far-UV circular dichroism (CD)

The CD spectra of N_TAIL_ proteins were recorded on a Jasco 810 dichrograph using 1-mm thick quartz cells in 10 mM sodium phosphate pH 7 at 20°C. CD spectra were measured between 190 and 260 nm, at 0.2 nm/min and are averages of three acquisitions. Mean ellipticity values per residue ([Θ]) were calculated as [Θ] = 3300 m ΔA/(l c n), where l (path length) = 0.1 cm, n = number of residues, m = molecular mass in daltons and c = protein concentration expressed in mg/mL. Number of residues (n) is 147, while m is 16 310 Da. Protein concentrations of 0.1 mg/mL were used when recording spectra. Structural variations of N_TAIL_ proteins were measured as a function of changes in the initial far-UV CD spectrum following addition of 20% 2,2,2 trifluoroethanol (TFE) (Sigma-Aldrich).

The experimental data in the 190–260 nm range were analyzed using the DICHROWEB website which was supported by grants to the BBSRC Centre for Protein and Membrane Structure and Dynamics [107,108]. The CDSSTR deconvolution method was used to estimate the content in α-helical and disordered structure using the reference protein set 7.

### Isothermal titration calorimetry

ITC experiments were carried out on an ITC200 isothermal titration calorimeter (Microcal) at 20° C. Protein pairs used in the binding analyses were dialyzed against the same buffer (10 mM Tris/HCl pH 8, 150 mM NaCl) to minimize undesirable buffer-related effects. The dialysis buffer was used in all preliminary equilibration and washing steps.

The concentrations of purified *wt* and mutated N_TAIL_ proteins in the microcalorimeter cell (0.2 mL) ranged from 25 μM to 180 μM. XD was added from a computer-controlled 40-μL microsyringe via a total of 19 injections of 2 μL each at intervals of 180 s. Its concentration in the microsyringe ranged from 300 μM to 960 μM.

A theoretical titration curve was fitted to the experimental data using the ORIGIN software (Microcal). This software uses the relationship between the heat generated by each injection and ΔH° (enthalpy change in kcal mole^-1^), K_A_ (association binding constant in M^-1^), n (number of binding sites per monomer), total protein concentration and free and total ligand concentrations. The variation in the entropy (ΔS° in cal mol^-1^ deg^-1^) of each binding reaction was inferred from the variation in the free energy (ΔG°), where this latter was calculated from the following relation: ΔG° = -RT *ln* 1/K_A_.

### Molecular dynamics simulations

All MD simulations were performed in explicit solvent with periodic conditions with CHARMM and NAMD software packages and CHARMM force field version 27 with CMAP corrections. The initial coordinates of the XD/α-MoRE complex were taken from the crystal structure (PDB code 1T6O) [44]. The two XD/α-MoRE mutated models bearing either the S491L or the R497G N_TAIL_ substitution, were built with VMD plugin ‘mutator’ starting from the X-ray structure of the *wt* complex (PDB code 1T6O). In the case of the S491L variant, the three most favourable orientations of the leucine side chain were generated with Sybyl. Non-protein derivatives were discarded. Orientation of the side chains of Asn, Gln, and His residues were checked using in-house VMD plugin and the WHAT IF web interface (http://swift.cmbi.kun.nl/). Residue His498 of XD was assigned HSD type and all other titratable groups were assigned standard protonation state at pH 7.0. Coordinates of missing hydrogen atoms were added using the hbuild algorithm in CHARMM. To improve conformational sampling, three independent simulations were carried out using different initial velocities. The system was solvated with a pre-equilibrated solvation box (edge length around 60 Å) consisting of TIP3P water molecules. Crystallographic water molecules were included in the initial model. Chloride and sodium ions were added to achieve neutralization of the whole system. Periodic boundary conditions were applied. Unfavorable contacts were removed by a short energy minimization with conjugate gradient and ABNR. Electrostatic interactions were treated using the particle-mesh Ewald summation method, and we used the switch function for the van der Waals energy interactions with cuton, cutoff and cutnb values of 10, 12 and 14 Å respectively. Vibration of the bonds containing hydrogen atoms were constrained with the Shake algorithm and a 1-fs integration step was used. The system was heated gradually to 300K, followed by an equilibration step (500 ps). During these two early steps, harmonic constraints were applied to protein heavy atoms. The constraint harmonic constant (k) was equal to 1 and 0.1 kcal/mol/Å^2^ for the backbone and side chains, respectively, and was removed after 250 ps equilibration. The production phase of 50 ns was performed without any constraints. Snapshots of the coordinates were saved every 0.5 ps. Trajectories were analyzed using a combination of in-house and VMD scripts.

### Analysis of molecular dynamics

Overall <RMSD> variations were computed with VMD after superimposition of the Cα atoms of each conformation generated onto the initial structure (last structure of the equilibration step). Flexible N- and C-terminal residues were not included in the calculation. Three types of RMSD were computed as it follows. For each frame, the XD protein was superimposed onto the initial XD model and RMSD was computed over XD Cα atoms only. For each frame the α–MoRE was superimposed onto the corresponding region of the initial structure and RMSD was computed over N_TAIL_ Cα atoms only. For each frame, the XD protein was superimposed onto the initial XD protein and RMSD was computed over N_TAIL_ Cα atoms only.

### Free energy perturbation

Free-energy perturbation (FEP) module implemented in NAMD was used to perform alchemical transformation of Ser491 to Leu and Arg497 to Gly. Free energies differences resulting from the Ser to Leu or Arg to Gly substitution were computed using the thermodynamic cycle shown in **S3 Fig**. The free form of the α–MoRE in solution was taken from the XD/N_TAIL_ complex.

### Free energy profile

The free energy profile for the dissociation of the XD/α-MoRE complex (*wt* and mutated forms) was computed using the adaptive biasing force (ABF) method, implemented in NAMD [109]. This method relies upon the integration of the average force acting on a selected reaction coordinate (here, the center of mass between the two partners). A biasing force is applied to the system in such a way that no average force acts along the reaction coordinate thus allowing overcoming free energy barriers. For a complete description of the method please refer to http://www.edam.uhp-nancy.fr/ABF/theory.html and references therein shown.

The distance separating the centers of mass of the two proteins was selected as reaction coordinate. The distance was calculated on the Cα atoms not taking into account the three atoms at each end (N-term and C-term end) of each protein partner due to their high flexibility. This distance is about 11 Å in the associated form and the partners are considered dissociated after a 10 Å increase in this distance. The reaction coordinate was subdivided into sections of 0.5 Å and each one was successively explored during 5 ns. Bin width was kept at 0.02 Å, the number of samples prior to force application was 500 and the wall force constant is 100 kcal.mol^-1^.Å^2^. Once a section is sampled, the conformation in which COM distance is the nearest to the upper boundary is selected as the starting point of the following 0.5 Å section. A post-processing step merges the sampling counts and the PMF of each part and generates the whole profile of PMF along the dissociation process. The trajectories were generated using the same protocol as described for free MD.

### Cell lines and viruses

Cells were cultured in DMEM medium (Life Technologies) supplemented with 10% of heat-inactivated (30 min at 56°C) fetal bovine serum, 1% L-glutamine, gentamicin (10 μg/ml) at 37°C and 5% CO_2_. Medium of 293-3-46 helper cells was supplemented with G418 at 1.2 mg/ml. Vero (si2) and Vero-SLAM (si1) cells stably expressing shRNA targeting the P and GFP mRNAs, respectively, were previously described [48]. To rescue recombinant viruses, the helper cell line 293-3-46 stably expressing T7 polymerase, MeV N, and P was transfected by using the ProFection kit with two plasmids coding for the MeV genome and MeV-L protein (pEMC-La) [110]. Three days after transfection, the cells were overlaid on either Vero (single N gene virus) or Vero-si2 cells (bi-N virus). Upon appearance, isolated syncytia were picked and individually propagated on relevant Vero (from CelluloNet BioBank BB-0033-00072, SFR BioSciences, Lyon France) (single N virus) or Vero-si2 (bi-N virus) cells. Virus stock was produced after a second passage at a multiplicity of infection (MOI) of 0.03 in the relevant cell line. This stock was checked to rule out mycoplasma contamination, has its N_1_, N_2_, P, M, and L genes sequenced, and was titrated on the relevant host cell before use.

### Split-luciferase reassembly assay

*Gaussia princeps* luciferase-based complementation assay and data analysis (normalized luminescent ratio, NLR) were performed according to [50]. Human 293T cells (from CelluloNet BioBank BB-0033-00072, SFR BioSciences, Lyon France) were cultured in Dulbecco's Medium Eagle’s Modified (DMEM) (Life Technologies) supplemented with 10% of heat inactivated (30 min at 56°C) fetal bovine serum, 1% L-Glutamine and 10 μg/ml gentamycin at 37°C and 5% CO2. Cells were transfected using the jetPRIME reagent (Polyplus transfection). NLR was calculated by dividing the luciferase value of the two chimeric partners by the sum of the luciferase value of every chimeric partner mixed with the other “empty” glu domain. Results were expressed as fold increase with respect to the reference N_TAIL_/XD, which was set to 1.

### Analysis of viral protein accumulation and virus replication

Parental Vero, si1 and si2 cells were infected at MOI 1 with recombinant viruses with or without addition of 10 μg/ml of fusion inhibitor peptide z-fFG to prevent syncytium formation. Virus production was measured after freeze-thaw cycles of infected cells using a 50% tissue culture infective dose (TCID50) titration assay. Contamination of virus stock with internal deletion and copyback defective interfering (DI) minigenomes were assessed according to the method of [111].

Detection of the expression of viral N, Flag-N1, HA-N2, P and cellular GAPDH proteins was performed by Western blotting. Infected cells were lysed in NP40 buffer (20 mM Tris/HCl pH 8, 150 mM NaCl, 0.6% NP-40, 2 mM EDTA, protease cocktail inhibitor Complete 1 x (Roche)) for 20 minutes on ice. The proteins were then separated from the cell debris by centrifugation at 15,000 g during 10 minutes. The proteins were denatured by the addition of Laemmli 1 x loading buffer before analysis by SDS-PAGE and immunoblotting using anti-N (cl25 antibody), anti-Flag (Sigma), anti-HA (Sigma), anti-P (49.21 antibody) and anti-GAPDH (Mab374, Chemicon) monoclonal antibodies. Western blotting was revealed by chemiluminescence as detailed previously [48].

Quantification of the MeV genome and mRNA contents of infected cells was performed by reverse transcription-quantitative PCR essentially as described previously [65], using the following primers. To quantify mRNA, sense N primer (5’-AAGAGATGGTAAGGAGGT-3’), antisense N primer (5’-ATGATACTTGGGCTTGTC-3’), sense P primer (5’-TGGACGGACCAGTTCCAGA-3’), antisense P primer (5’-GGCTCCTTTGATATCATCAAG-3’), sense F primer (5’-GCTCAGATAACAGCCGGCATT-3’), antisense F primer (5’-AGCTTCTGGCCGATTA-3’) were used. Negative-strand genome was reverse transcribed using sense 5’-tagged N primer (5’-gcagggcaatctcacaatcaggAAGAGATGGTAAGGAGGT-3’), and the cDNA was PCR quantified using sense tag primer (5’-gcagggcaatctcacaatcagg-3’) and antisense N primer. For the genome the results were expressed as copy number/μg RNA, and for transcripts the results were expressed either as the number of polymerized nucleotides/genome copy or as the viral transcript/μg RNA after normalization for the genome copy contents of each sample.

### Minigenome assay

The assay was performed essentially as described in [62,112] with minor modifications. 2.10^4^ BSRT7 cells that constitutively express the T7 phage DNA-dependent RNA polymerase [113] were seeded in 96-well plates and transfected the day after with 66 ng of pEMC-N (either *wt* or mutated) 44 ng of pEMC-(Flag/L+P) (a home-made T7-driven bicistronic construct) [58] and 90 ng of plasmid encoding for the different minigenomes mixed with the transfection reagent as indicated in the manufacturer protocol (jetPRIME Polyplus-transfection). Two days after transfection, Firefly and NanoLuc activity were measured using the Nano-Glo Dual-Luciferase Reporter Assay (Promega). The background luciferase activity from of both luciferases observed in the absence of active L protein was subtracted from the signal measured in the presence of L, and data obtained from three independent experiments were normalized to each other to level the mean signal observed for all combinations tested at the same time. The percentage of unpriming per nucleotide of the un-transcribed intergenic (UTIGR) region (%unpriming/^UTIGR^nt) was calculated as follow. The luciferase signals ratios were plotted in relation to the length of the UTIGR region and the equation of the exponential regression curve was calculated (y = b*e^a^). The %unpriming/^UTIGR^nt = 1-e^a^.

## Supporting Information

S1 Fig Protein expression of glu1-N variants 293T cells were transfected one day before with each construct and lysed in 0.6% NP40and 6M Urea buffer. Extracts were electrophoresed on a 12% bis-acrylamide gel and proteins were electro-transferred and proteins detected with antibodies cl25 anti-N and anti-GAPDH monoclonal antibodies.
(PDF)

S2 Fig Hydrophobic residue contacts and major hydrogen bonds across the N_TAIL_/XD protein-protein interface during the time course of molecular dynamics trajectories. (**a**) Wild type N_TAIL_/XD complex. (**b**). S491L N_TAIL_ variant. (**c**). R497G N_TAIL_ variant. Hydrogen bonds summarized in Table 1 are highlighted as grey dotted lines. Numbers indicate the occurrence of each hydrogen bond during the simulations as a percentage of time. XD and N_TAIL_ residues are labeled in pink and green, respectively. This figure was generated with LigPlot+ program for automatic generation of 2D protein-ligand and protein-protein interaction diagrams (http://www.ebi.ac.uk/thornton-srv/software/LigPlus/) [114].
(PDF)

S3 Fig Thermodynamic cycle used in FEP to calculate the relative binding energies resulting from Ser to Leu or Arg to Gly substitutions. (PDF)

S4 Fig **Improved signal ratio of edited NanoLuc gene over Firefly gene upon transcription by P+L MeV polymerase** with (**a**) raw data and (**b**) improved dynamic range. Data were obtained using dual-luciferase minigenome with Firefly and edited NanoLuc gene separated by canonical N-P IGRs in the presence of *wt*, N, P and L or a truncated inactive L (L^ko^) as a negative control to measure the translation background from the minigenome transcribed by the T7 polymerase. The level of the background RLU signal is shown by the grey zone in (**a**)
(PDF)

S5 Fig Homogenous Firefly signals observed when assessing the ability of N_TAIL_ variants to support gene reporter expression from dual-luciferase minigenomes with each MeV IGR. **(a)** Firefly signals observed for each N variant/minigenome combination ranked by N variant (top right) with mean value for all variants (top left) and by minigenome (bottom). **(b)** Absence of correlation between Firefly signals observed with each N_TAIL_ variant and binding strength to XD.
(PDF)

S6 Fig **(a)** Parameters known to affect MeV transcription rate of each gene and transcription gradient (see also Plumet *et al* [65]. **(b)** RNA synthesis parameters that are affected by the affinity between N_TAIL_ and XD according to Brunel *et al* [48] for RNA synthesis rate and according to this work for the efficiency in scanning and/or re-initiation.
(PDF)

S7 Fig Homogenous Firefly signals observed when assessing the ability of N_TAIL_ variants to support gene reporter expression from dual-luciferase minigenomes with elongated UTIGR. **(a)** Firefly signals observed for each N variant/minigenome combination ranked by N variant (top right) with mean value for all variants (top left) and by minigenome (bottom). **(b)** Absence of correlation between Firefly signals observed with each N_TAIL_ variant and binding strength to XD.
(PDF)

S8 Fig Principle of biG-biS MeV viruses selective expression of the duplicated N1 and N2 gene according to the Vero cell host (see [48] for details). (PDF)

S9 Fig **Characterization of recombinant unigene MeV expressing N**_**TAIL**_ **variants** (**a**) Western blot analysis of N and P expression in Vero cells infected by recombinant unigene viruses. (**b-d**) lack of detectable contamination by internally deleted (**b**) or copyback (**c**) DIs and (**d**) genome of each recombinant virus as detected by RT-PCR (see [48] for method).
(PDF)

S10 Fig Ability of equal mixture of *wt* N and D493G variant to support reporter genes from N-P IGR 2-gene minigenome. Minigenome data are expressed as the mean +/- SD of 2 independent experiments, with each combination being done in triplicate. See Fig 6A for minigenome structure.
(PDF)

S11 Fig Characterization of recombinant unigene MeV expressing N_TAIL_ variants. Correlation between viral RNA measurements in cells infected by the unigene viruses with accumulation rate of N (+) as a function of accumulation rate of P(+) RNA during primary transcription at early times post-infection (see [65] for temporal windows) **(a)**, N(+) RNA as function of P(+) RNA levels at 24 h.p.i. **(b)**, N(+) **(c)** and P(+) **(d)** RNA levels as function of genomic (-) RNA levels measured at 24 h.p.i.
(PDF)

S12 Fig **Ability of truncated N1-439 in comparison with *wt* N and R497G variant to support transcription and re-initiation over elongated UTIGR (a)** Firefly signals from dual-luciferase minigenomes with elongated UTIGR. **(b)** Re-initiation efficiency at the second gene with statistical significance when compared to wt N efficiency, * p<0.05, ** p<0.02 & below, ns p = 0.38.
(PDF)

S1 Table Average and standard deviation of root mean square deviation (RMSD) over CA atoms during the whole 50 ns trajectories (values are in Angstrom). The values are the result of at least 2 trajectories. ^a^ XD domain of all MD structures was superimposed on the initial XD domain and RMSD values were computed for the XD domain. ^b^ NTAIL α-helix of all MD structures was superimposed on the initial NTAIL helix and RMSD values were computed for NTAIL atoms. ^c^ XD domain of all MD structures was superimposed on the initial XD domain and RMSD values were computed for the NTAIL helix.
(PDF)

S2 Table DNA (+) sequence of the Firefly/NanoLuc 2-gene minigenome with conditional expression of NanoLuc to RNA edition. Firefly and NanoLuc luciferase ORFs are in capital letters. The P editing site in underlined in yellow.
(PDF)

S3 Table DNA (+) sequences of elongated N-P IGR added in minigenomes. (PDF)

S4 Table DNA (+) sequence of the 3-gene minigenome used for the measurement of read-through transcripts. Coding sequences are in capital letter.
(PDF)
