# Supplementary material for: Modulation of Re-initiation of Measles Virus Transcription at Intergenic Regions by PXD to NTAIL Binding Strength
Source: PLoS Pathog. 2016 Dec 9;12(12):e1006058. doi: 10.1371/journal.ppat.1006058 (PMC5148173; doi:10.1371/journal.ppat.1006058)
Supplement: S1 Fig — Extracts were electrophoresed on a 12% bis-acrylamide gel and proteins were electro-transferred and proteins detected with antibodies cl25 anti-N and anti-GAPDH monoclonal antibodies. (PDF) [file ppat.1006058.s001.pdf]

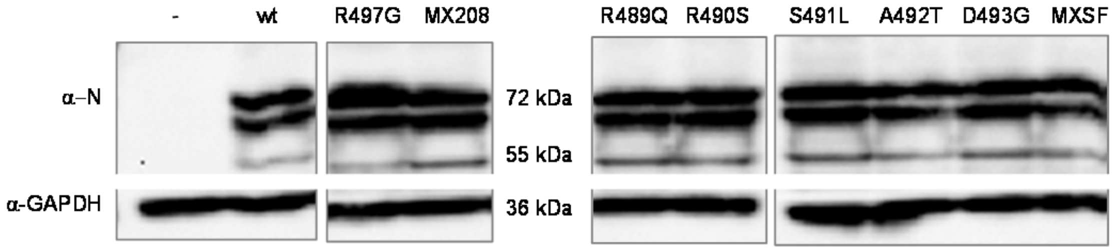

S1 Fig. Protein expression of glu1-N variants 293T cells were transfected one day before with each construct and lysed in 0.6% NP40and 6M Urea buffer. Extracts were electrophoresed on a 12% bis-acrylamide gel and proteins were electro-transferred and proteins detected with antibodies cl25 anti-N and anti-GAPDH monoclonal antibodies.
