# Supplementary material for: Modulation of Re-initiation of Measles Virus Transcription at Intergenic Regions by PXD to NTAIL Binding Strength
Source: PLoS Pathog. 2016 Dec 9;12(12):e1006058. doi: 10.1371/journal.ppat.1006058 (PMC5148173; doi:10.1371/journal.ppat.1006058)
Supplement: S2 Fig — (a) Wild type NTAIL/XD complex. (b). S491L NTAIL variant. (c). R497G NTAIL variant. Hydrogen bonds summarized in Table 1 are highlighted as grey dotted lines. Numbers indicate the occurrence of each hydrogen bond during the simulations as a percentage of time. XD and NTAIL residues are labeled in pink and green, respectively. This figure was generated with LigPlot+ program for automatic generation of 2D protein-ligand and protein-protein interaction diagrams (http://www.ebi.ac.uk/thornton-srv/software/LigPlus/) [114]. (PDF) [file ppat.1006058.s002.pdf]

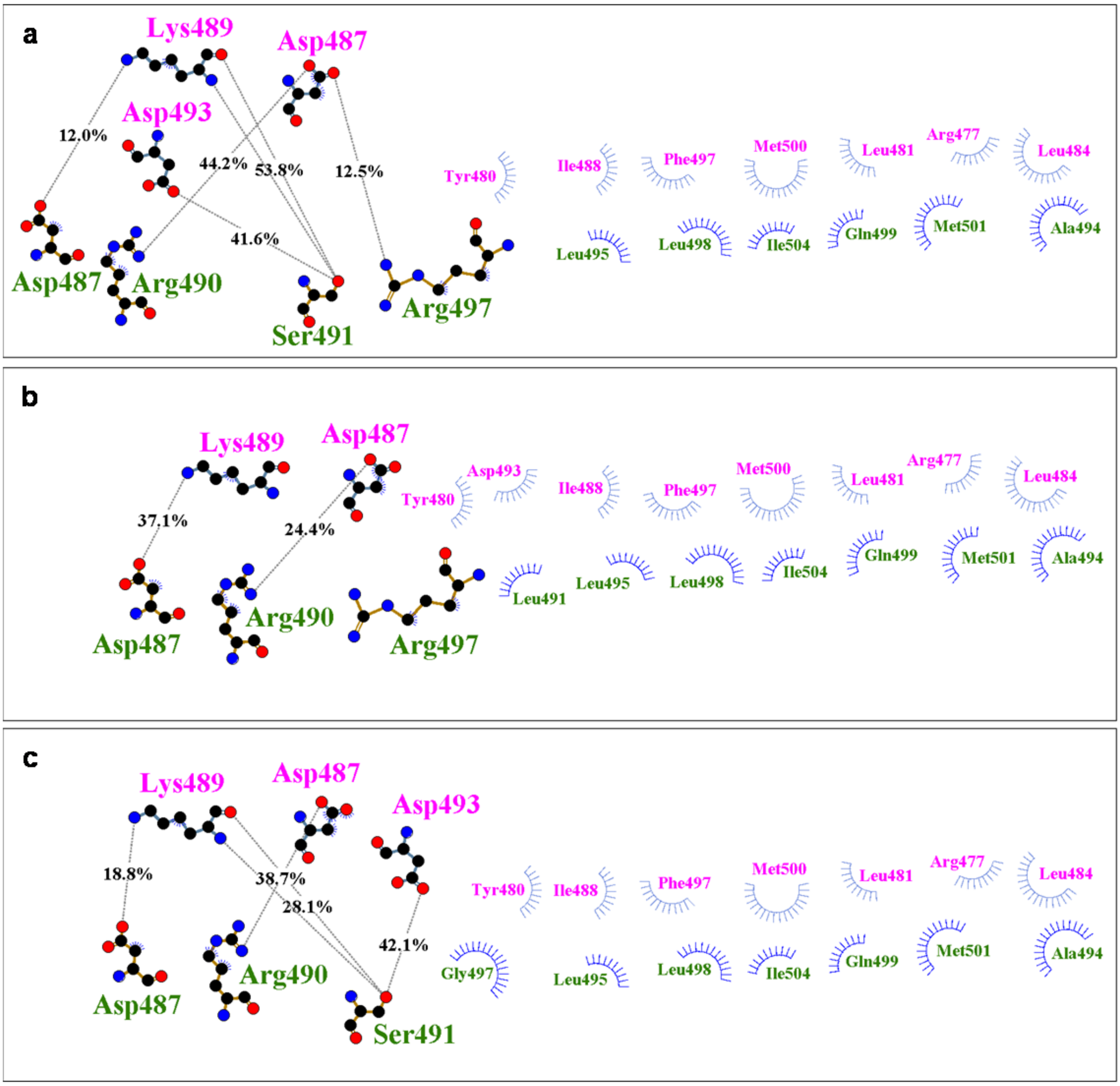

S2 Fig. Hydrophobic residue contacts and major hydrogen bonds across the N_{TAIL}/XD protein-protein interface during the time course of molecular dynamics trajectories. (**a**) Wild type N_{TAIL}/XD complex. (**b**). S491L N_{TAIL} mutant. (**c**). R497G N_{TAIL} mutant. Hydrogen bonds summarized in Table 1 are highlighted as grey dotted lines. Numbers indicate the occurrence of each hydrogen bond during the simulations as a percentage of time. XD and N_{TAIL} residues are labeled in pink and green, respectively. This figure was generated with LigPlot+ program for automatic generation of 2D protein-ligand and protein-protein interaction diagrams (http://www.ebi.ac.uk/thornton-srv/software/LigPlus/) [1].

Reference

1. Laskowski RA, Swindells MB (2011) LigPlot+: multiple ligand-protein interaction diagrams for drug discovery. J Chem Inf Model 51: 2778-2786.
