# Supplementary material for: Modulation of Re-initiation of Measles Virus Transcription at Intergenic Regions by PXD to NTAIL Binding Strength
Source: PLoS Pathog. 2016 Dec 9;12(12):e1006058. doi: 10.1371/journal.ppat.1006058 (PMC5148173; doi:10.1371/journal.ppat.1006058)
Supplement: S3 Fig — (PDF) [file ppat.1006058.s003.pdf]

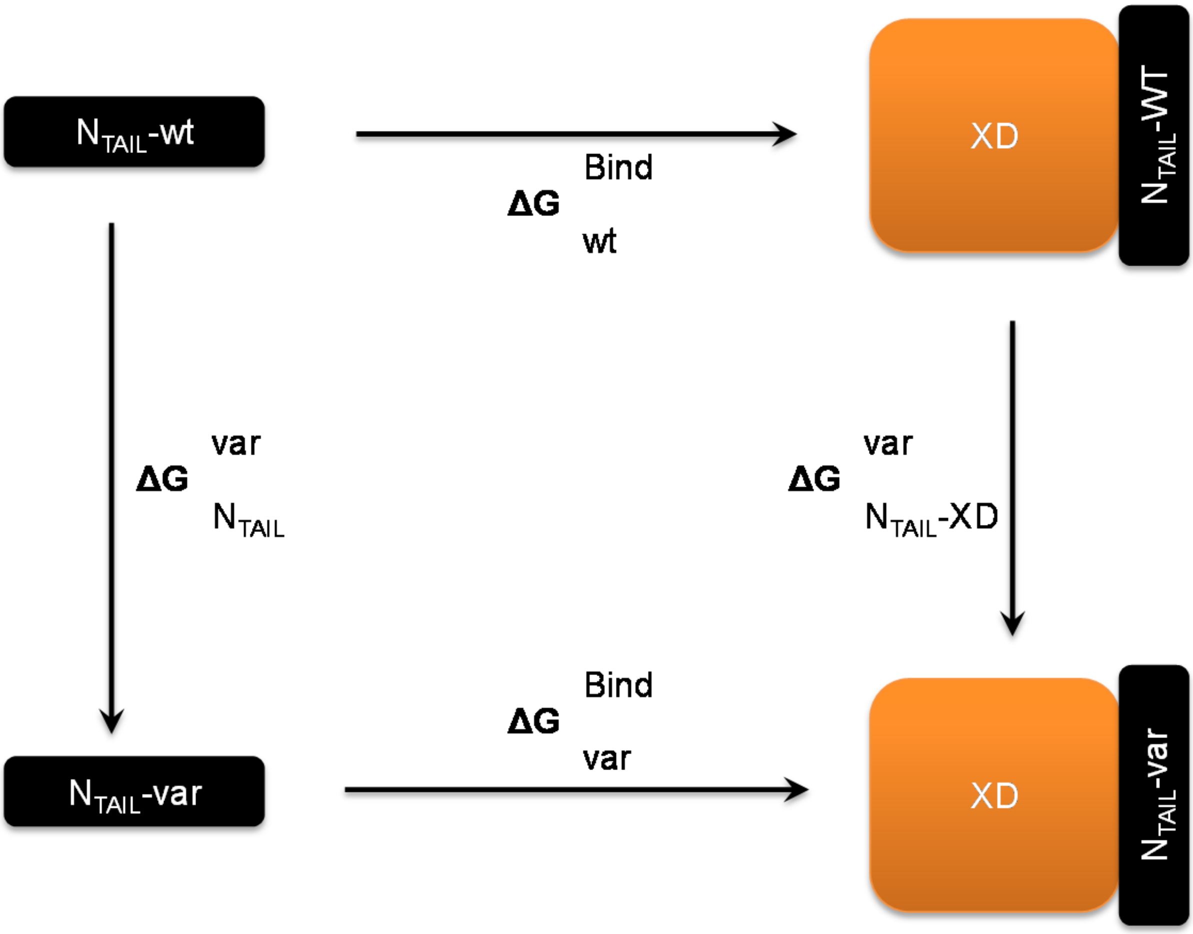

S3 Fig. Thermodynamic cycle used in FEP to calculate the relative binding energies resulting from Ser to Leu or Arg to Gly substitutions.
