# Supplementary material for: Modulation of Re-initiation of Measles Virus Transcription at Intergenic Regions by PXD to NTAIL Binding Strength
Source: PLoS Pathog. 2016 Dec 9;12(12):e1006058. doi: 10.1371/journal.ppat.1006058 (PMC5148173; doi:10.1371/journal.ppat.1006058)
Supplement: S4 Fig — Improved signal ratio of edited NanoLuc gene over Firefly gene upon transcription by P+L MeV polymerase with (a) raw data and (b) improved dynamic range. Data were obtained using dual-luciferase minigenome with Firefly and edited NanoLuc gene separated by canonical N-P IGRs in the presence of wt, N, P and L or a truncated inactive L (Lko) as a negative control to measure the translation background from the minigenome transcribed by the T7 polymerase. The level of the background RLU signal is shown by the grey zone in (a) (PDF) [file ppat.1006058.s004.pdf]

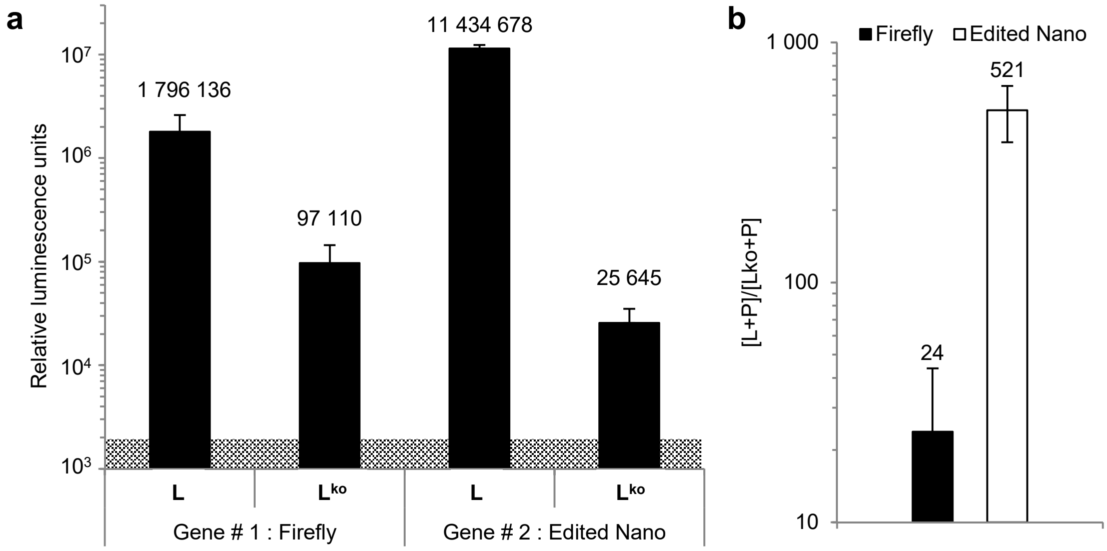

S4 Fig. Improved signal ratio of edited Nano gene over Firefly gene upon transcription by P+L MeV polymerase with (**a**) raw data and (**b**) improved dynamic range. Data were obtained using dual-luciferase minigenome with Firefly and edited Nano gene separated by canonical N-P IGRs in the presence of *wt*, N, P and L or a truncated inactive L (L^{ko}) as a negative control to measure the translation background from the minigenome transcribed by the T7 polymerase. The level of the background RLU signal is shown by the grey zone in (**a**)
