# Supplementary material for: Modulation of Re-initiation of Measles Virus Transcription at Intergenic Regions by PXD to NTAIL Binding Strength
Source: PLoS Pathog. 2016 Dec 9;12(12):e1006058. doi: 10.1371/journal.ppat.1006058 (PMC5148173; doi:10.1371/journal.ppat.1006058)
Supplement: S6 Fig — (a) Parameters known to affect MeV transcription rate of each gene and transcription gradient (see also Plumet et al [65]. (b) RNA synthesis parameters that are affected by the affinity between NTAIL and XD according to Brunel et al [48] for RNA synthesis rate and according to this work for the efficiency in scanning and/or re-initiation. (PDF) [file ppat.1006058.s006.pdf]

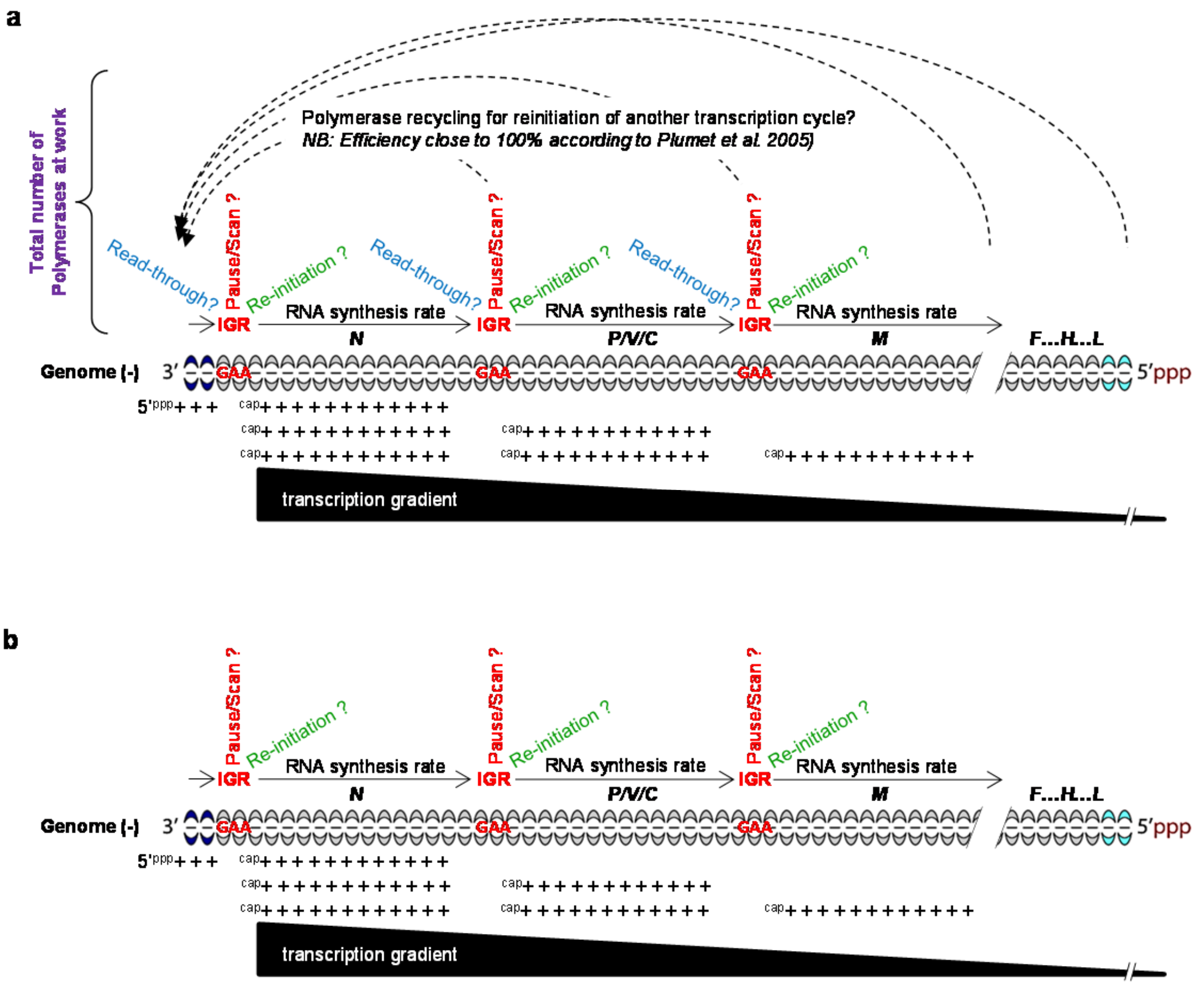

S6 Fig. **(a)** Parameters known to affect MeV transcription rate of each gene and transcription gradient (see also Plumet *et al* [1]. **(b)** RNA synthesis parameters that are affected by the affinity between N_{TAIL} and XD according to Brunel *et al* [2]for RNA synthesis rate and according to this work for the efficiency in scanning and/or re-initiation.

1. Plumet S, Duprex WP, Gerlier D (2005) Dynamics of viral RNA synthesis during measles virus infection. Journal of Virology 79: 6900-6908.

2. Brunel J, Chopy D, Dosnon M, Bloyet LM, Devaux P, et al. (2014) Sequence of events in measles virus replication: role of phosphoprotein-nucleocapsid interactions. Journal of virology 88: 10851-10863.
