# Supplementary material for: Modulation of Re-initiation of Measles Virus Transcription at Intergenic Regions by PXD to NTAIL Binding Strength
Source: PLoS Pathog. 2016 Dec 9;12(12):e1006058. doi: 10.1371/journal.ppat.1006058 (PMC5148173; doi:10.1371/journal.ppat.1006058)
Supplement: S7 Fig — (a) Firefly signals observed for each N variant/minigenome combination ranked by N variant (top right) with mean value for all variants (top left) and by minigenome (bottom). (b) Absence of correlation between Firefly signals observed with each NTAIL variant and binding strength to XD. (PDF) [file ppat.1006058.s007.pdf]

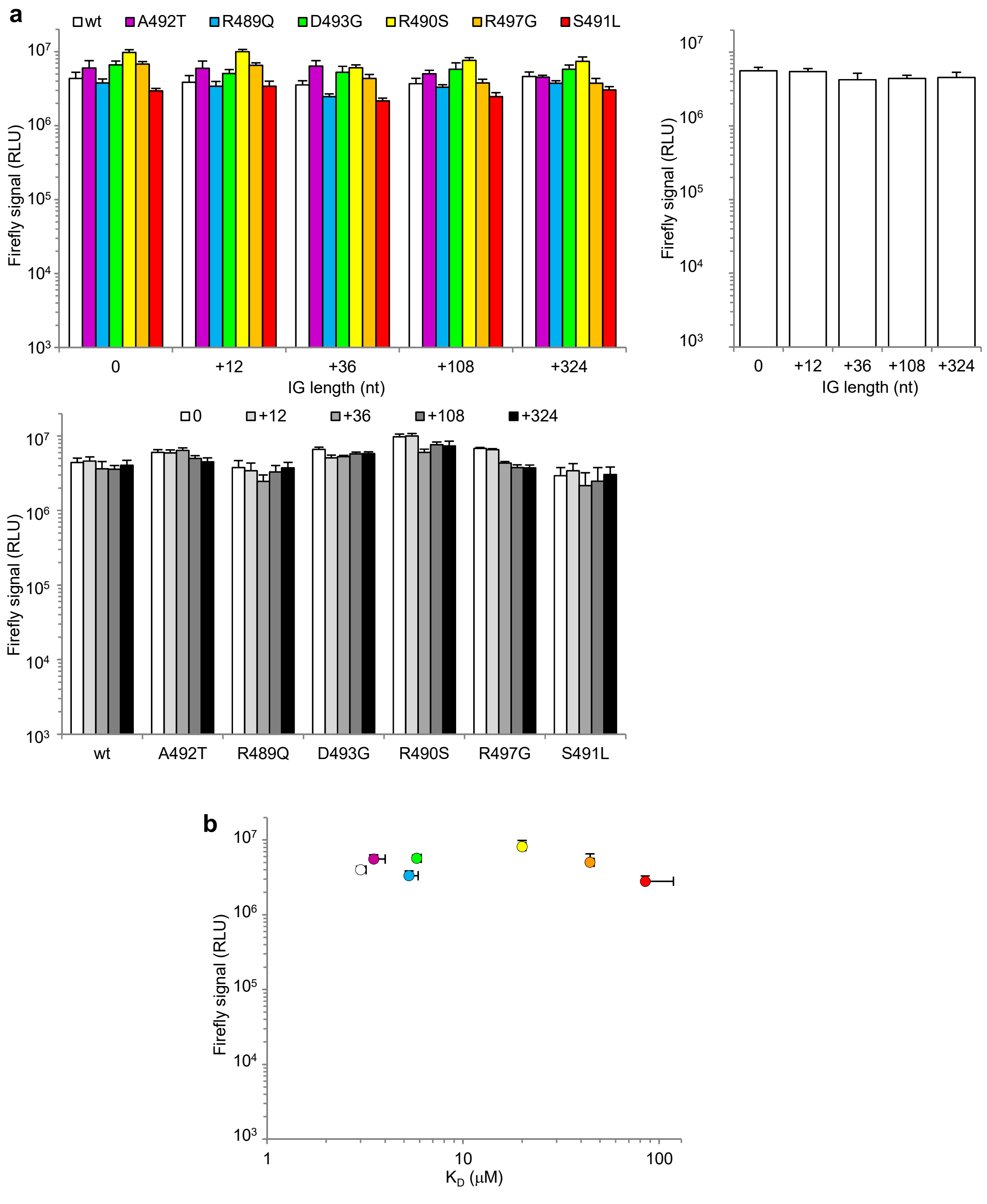

S7 Fig. Homogenous Firefly signals observed when assessing the ability of N_{TAIL} variants to support gene reporter expression from dual-luciferase minigenomes with elongated UTIGR. (a) Firefly signals observed for each N variant/minigenome combination ranked by N variant (top right) with mean value for all variants (top left) and by minigenome (bottom). **(b)** Absence of correlation between Firefly signals observed with each N_{TAIL} variant and binding strength to XD.
