# Supplementary material for: Modulation of Re-initiation of Measles Virus Transcription at Intergenic Regions by PXD to NTAIL Binding Strength
Source: PLoS Pathog. 2016 Dec 9;12(12):e1006058. doi: 10.1371/journal.ppat.1006058 (PMC5148173; doi:10.1371/journal.ppat.1006058)
Supplement: S8 Fig — (PDF) [file ppat.1006058.s008.pdf]

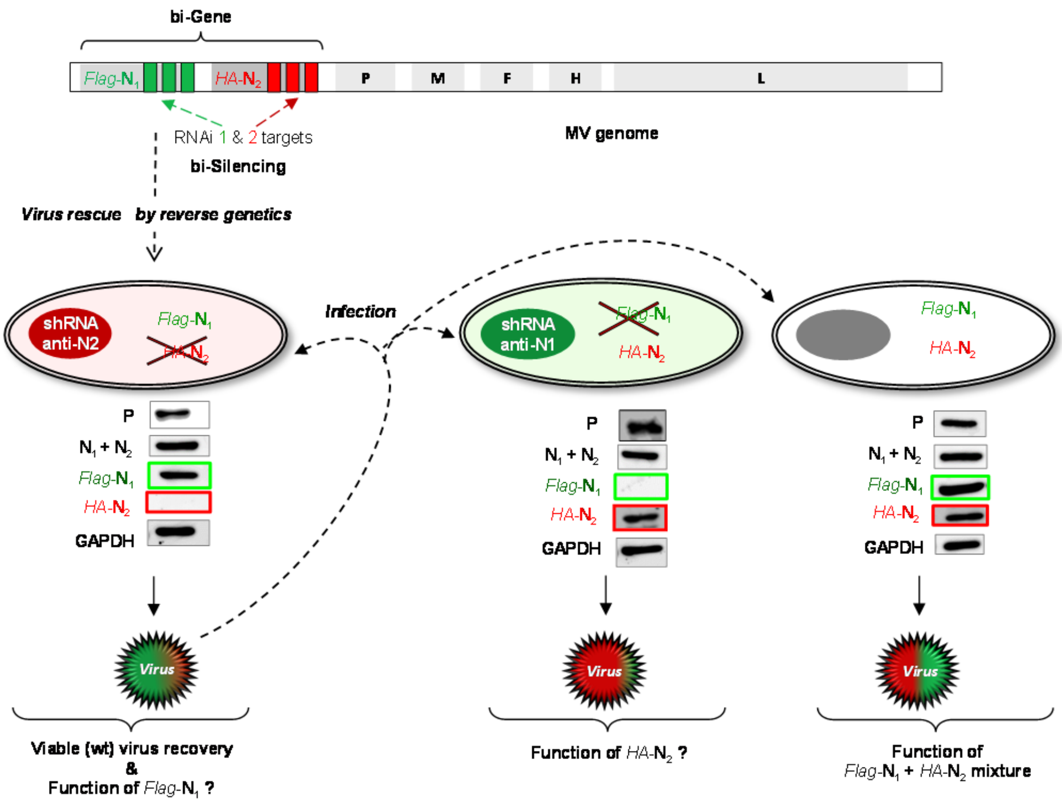

S8 Fig. Principle of biG-biS MeV viruses selective expression of the duplicated N1 and N2 gene according to the Vero cell host (see [3] for details).
