# Supplementary material for: Modulation of Re-initiation of Measles Virus Transcription at Intergenic Regions by PXD to NTAIL Binding Strength
Source: PLoS Pathog. 2016 Dec 9;12(12):e1006058. doi: 10.1371/journal.ppat.1006058 (PMC5148173; doi:10.1371/journal.ppat.1006058)
Supplement: S9 Fig — Characterization of recombinant unigene MeV expressing N TAIL variants (a) Western blot analysis of N and P expression in Vero cells infected by recombinant unigene viruses. (b-d) lack of detectable contamination by internally deleted (b) or copyback (c) DIs and (d) genome of each recombinant virus as detected by RT-PCR (see [48] for method). (PDF) [file ppat.1006058.s009.pdf]

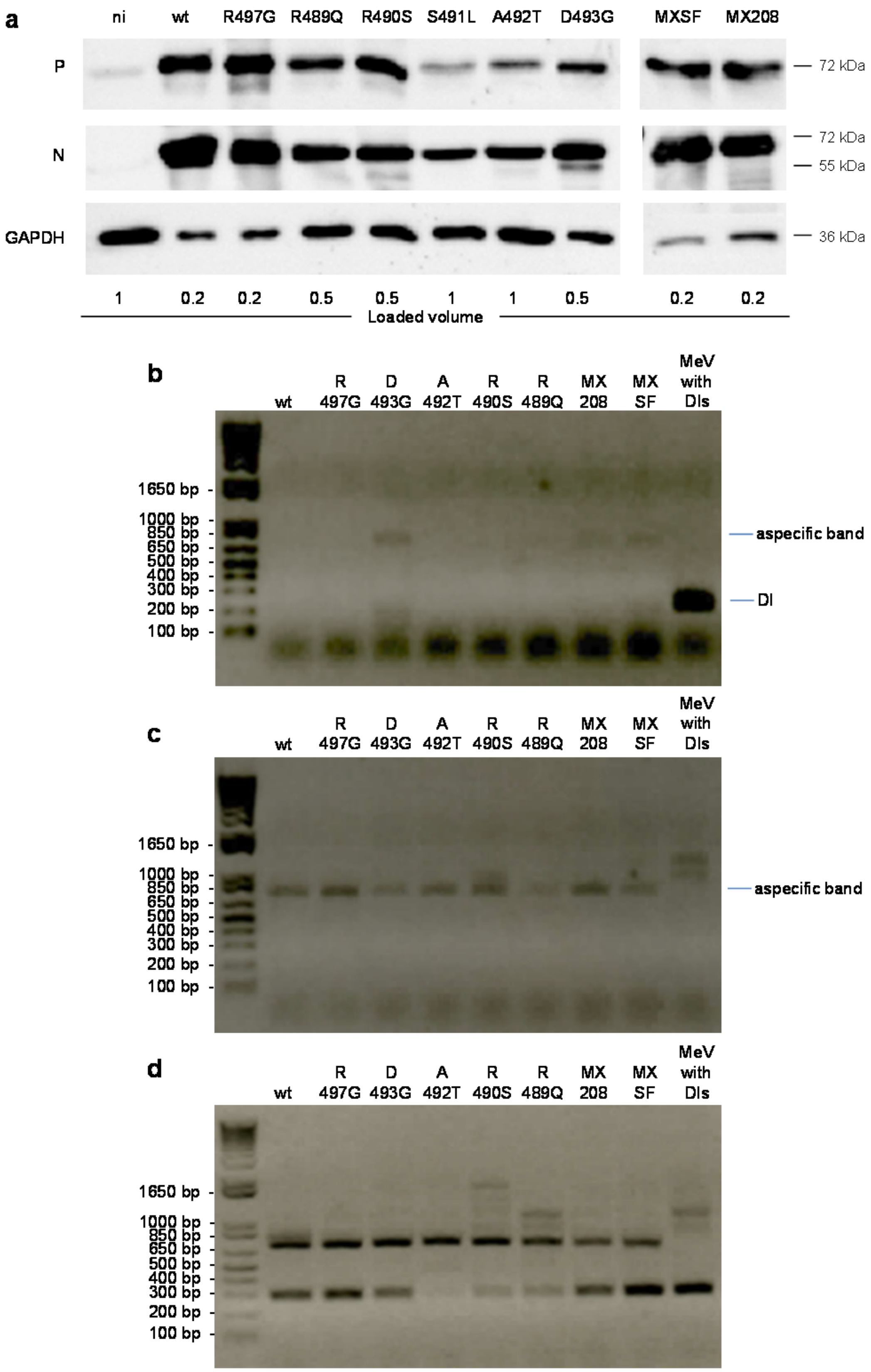

S9 Fig. Characterization of recombinant unigene MeV expressing N_{TAIL} variants (**a**) Western blot analysis of N and P expression in Vero cells infected by recombinant unigene viruses. (**b-d**) lack of detectable contamination by internally deleted (**b**) or copyback (**c**) DIs and (**d**) genome of each recombinant virus as detected by RT-PCR (see [1] for method).

1. Brunel J, Chopy D, Dosnon M, Bloyet LM, Devaux P, et al. (2014) Sequence of events in measles virus replication: role of phosphoprotein-nucleocapsid interactions. Journal of virology 88: 10851-10863.
