# Supplementary material for: Modulation of Re-initiation of Measles Virus Transcription at Intergenic Regions by PXD to NTAIL Binding Strength
Source: PLoS Pathog. 2016 Dec 9;12(12):e1006058. doi: 10.1371/journal.ppat.1006058 (PMC5148173; doi:10.1371/journal.ppat.1006058)
Supplement: S10 Fig — Minigenome data are expressed as the mean +/- SD of 2 independent experiments, with each combination being done in triplicate. See Fig 6A for minigenome structure. (PDF) [file ppat.1006058.s010.pdf]

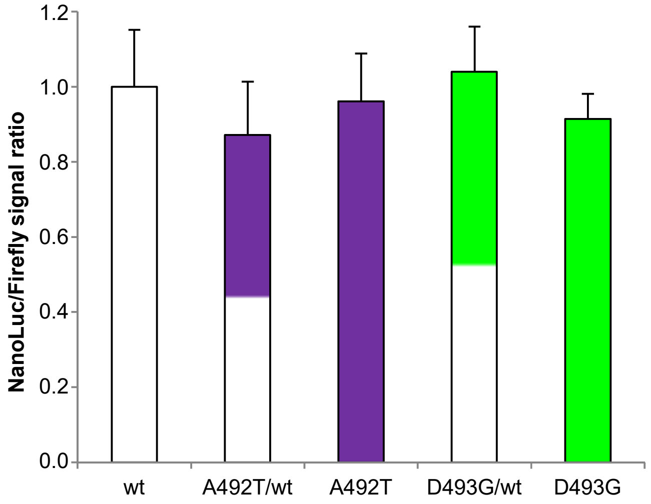

S10 Fig. Ability of equal mixture of *wt* N and D493G variant to support reporter genes from N-P IGR minigenome. Minigenome data are expressed as the mean +/- SD of 2 independent experiments, with each combination being done in triplicate. See Fig. 6A for minigenome structure.
