# Supplementary material for: Modulation of Re-initiation of Measles Virus Transcription at Intergenic Regions by PXD to NTAIL Binding Strength
Source: PLoS Pathog. 2016 Dec 9;12(12):e1006058. doi: 10.1371/journal.ppat.1006058 (PMC5148173; doi:10.1371/journal.ppat.1006058)
Supplement: S11 Fig — Correlation between viral RNA measurements in cells infected by the unigene viruses with accumulation rate of N (+) as a function of accumulation rate of P(+) RNA during primary transcription at early times post-infection (see [65] for temporal windows) (a), N(+) RNA as function of P(+) RNA levels at 24 h.p.i. (b), N(+) (c) and P(+) (d) RNA levels as function of genomic (-) RNA levels measured at 24 h.p.i. (PDF) [file ppat.1006058.s011.pdf]

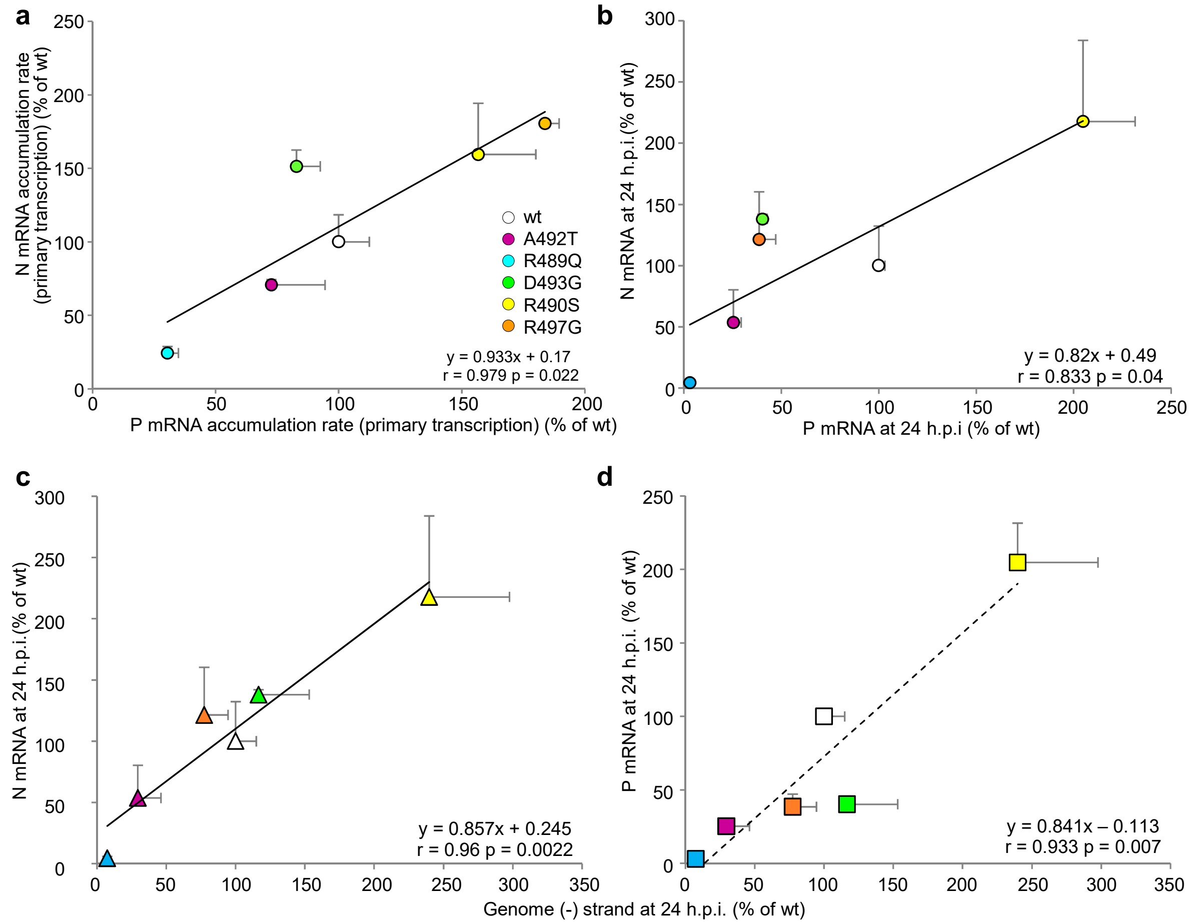


**S11 Fig. Characterization of recombinant unigene MeV expressing $N_{TAIL}$ variants.** Correlation between viral RNA measurements in cells infected by the unigene viruses with accumulation rate of N (+) as a function of accumulation rate of P(+) RNA during primary transcription at early times post-infection (see [2] for temporal windows) (a), N(+) RNA as function of P(+) RNA levels at 24 h.p.i. (b), N(+) (c) and P(+) (d) RNA levels as function of genomic (-) RNA levels measured at 24 h.p.i.
