# Supplementary material for: Modulation of Re-initiation of Measles Virus Transcription at Intergenic Regions by PXD to NTAIL Binding Strength
Source: PLoS Pathog. 2016 Dec 9;12(12):e1006058. doi: 10.1371/journal.ppat.1006058 (PMC5148173; doi:10.1371/journal.ppat.1006058)
Supplement: S12 Fig — Ability of truncated N1-439 in comparison with wt N and R497G variant to support transcription and re-initiation over elongated UTIGR (a) Firefly signals from dual-luciferase minigenomes with elongated UTIGR. (b) Re-initiation efficiency at the second gene with statistical significance when compared to wt N efficiency, * p<0.05, ** p<0.02 & below, ns p = 0.38. (PDF) [file ppat.1006058.s012.pdf]

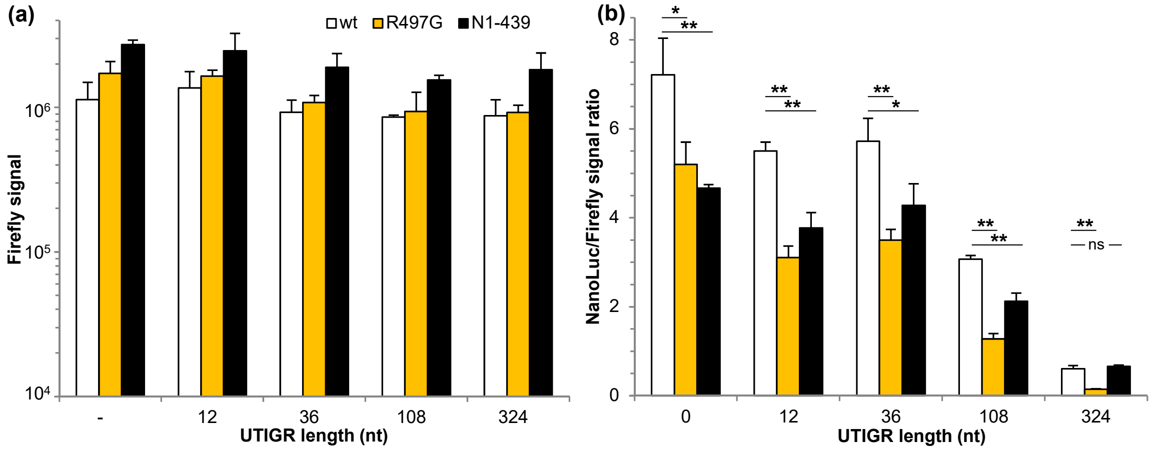

S12 Fig. Ability of truncated N1-439 in comparison with *wt* N and R497G variant to support transcription and reinitiation over elongated UTIGR (a) Firefly signals from dual-luciferase minigenomes with elongated UTIGR. **(b)** Re-initiation efficiency at the second gene with statistical significance when compared to *wt* N efficiency, * $p<0.05$, ** $p<0.02$ & below, ns $p=0.38$.
