# Supplementary material for: Modulation of Re-initiation of Measles Virus Transcription at Intergenic Regions by PXD to NTAIL Binding Strength
Source: PLoS Pathog. 2016 Dec 9;12(12):e1006058. doi: 10.1371/journal.ppat.1006058 (PMC5148173; doi:10.1371/journal.ppat.1006058)
Supplement: S1 Table — The values are the result of at least 2 trajectories. a XD domain of all MD structures was superimposed on the initial XD domain and RMSD values were computed for the XD domain. b NTAIL α-helix of all MD structures was superimposed on the initial NTAIL helix and RMSD values were computed for NTAIL atoms. c XD domain of all MD structures was superimposed on the initial XD domain and RMSD values were computed for the NTAIL helix. (PDF) [file ppat.1006058.s013.pdf]

| | XD[a] | $N_{TAIL}$[b] | $N_{TAIL}$ vs XD[c] |
|---|---|---|---|
| ***wt*** | 0.77 ± 0.15 | 0.52 ± 0.13 | 1.01 ± 0.33 |
| **S491L** | 0.77 ± 0.13 | 0.42 ± 0.11 | 1.19 ± 0.34 |
| **R497G** | 0.73 ± 0.12 | 0.45 ± 0.11 | 1.15 ± 0.37 |

**S1 Table :** Average and standard deviation of root mean square deviation (RMSD) over CA atoms during the whole 50 ns trajectories (values are in Angstrom). The values are the result of at least 2 trajectories. [a] XD domain of all MD structures was superimposed on the initial XD domain and RMSD values were computed for the XD domain. [b] $N_{TAIL}$ $\alpha$-helix of all MD structures was superimposed on the initial $N_{TAIL}$ helix and RMSD values were computed for $N_{TAIL}$ atoms. [c] XD domain of all MD structures was superimposed on the initial XD domain and RMSD values were computed for the $N_{TAIL}$ helix.
