# Supplementary material for: Modulation of Re-initiation of Measles Virus Transcription at Intergenic Regions by PXD to NTAIL Binding Strength
Source: PLoS Pathog. 2016 Dec 9;12(12):e1006058. doi: 10.1371/journal.ppat.1006058 (PMC5148173; doi:10.1371/journal.ppat.1006058)
Supplement: S3 Table — (PDF) [file ppat.1006058.s015.pdf]

S3 Table. DNA (+) sequences of elongated N-P IGR added in minigenomes.

N-P minigenome from N-stop to P-ATG.

TAAgtgcgagaggccgagggccagaacaacatccgcctaccatccatcattgttataaaaaaCTTaggaaccaggtccacacagccgccagcccatcaaccatccactcccacgattggagccaATG

N-P minigenome elongated by 12 nt

TAAgtgcgagaggccgagggccagaacaacatccgcctaccatccatcattgttataaaaaaCtacGtaaaaaaCTTaggaaccaggtccacacagccgccagcccatcaaccatccactcccacgattggagccaATG

N-P minigenome elongated by 36 nt

TAAgtgcgagaggccgagggccagaacaacatccgcctaccatccatcattgttataaaaaaCtactgagactacatcagctattctgatGtaaaaaaCTTaggaaccaggtccacacagccgccagcccatcaaccatccactcccacgattggagccaATG

N-P minigenome elongated by 108 nt

TAAgtgcgagaggccgagggccagaacaacatccgcctaccatccatcattgttataaaaaaCtactgagactacatcagctattctgattacacccgaggggggatgataaaccgggcgcggtcggtaaagttgttccatttttttgaagcgaaggttgtggaGtaaaaaaCTTaggaaccaggtccacacagccgccagcccatcaaccatccactcccacgattggagccaATG

N-P minigenome elongated by 324 nt

TAAgtgcgagaggccgagggccagaacaacatccgcctaccatccatcattgttataaaaaaCtactgagactacatcagctattctgattacacccgaggggggatgataaaccgggcgcggtcggtaaagttgttccatttttttgaagcgaaggttgtggatctggataccgggaaaacgctgggcgttaatcagagaggcgaattatgtgtcagaggacctatgattatgtccggttatgtaaacaatccggaagcgaccaacgccttgattgacaaggatggatggctacattctggagacatagcttactgggacgaagacgaacacttcttcatagttgaccgcttgaagtctttaattaaatacaaaggataGtaaaaaaCTTaggaaccaggtccacacagccgccagcccatcaaccatccactcccacgattggagccaATG
