# Supplementary material for: Modulation of Re-initiation of Measles Virus Transcription at Intergenic Regions by PXD to NTAIL Binding Strength
Source: PLoS Pathog. 2016 Dec 9;12(12):e1006058. doi: 10.1371/journal.ppat.1006058 (PMC5148173; doi:10.1371/journal.ppat.1006058)
Supplement: S4 Table — Coding sequences are in capital letter. (PDF) [file ppat.1006058.s016.pdf]

S4 Table. DNA (+) sequence of the 3-gene minigenome used for the measurement of read-through transcripts. Coding sequences are in capital letter.

accaaacaaagttgggtaaggatagttcaatcaatgatcatcttctagtgca**CTT**aggattcaagatcctattatcagggacaagagcaggattagggatatccga
g**ATG**GAAGACGCCAAAAACATAAAGAAAGGCCCGGCGCCATTCTATCCGCTGGAAGATGGAACCG
CTGGAGAGCAACTGCATAAGGCTATGAAGAGATACGCCCTGGTTCCTGGAACAATTGCTTTTACAG
ATGCACATATCGAGGTGGACATCACTTACGCTGAGTACTTCGAAATGTCCGTTCGGTTGGCAGAAG
CTATGAAACGATATGGGCTGAATACAAATCACAGAATCGTCGTATGCAGTGAAAACTCTCTTCAAT
TCTTTATGCCGGTGTTGGGCGCGTTATTTATCGGAGTTGCAGTTGCGCCCGCGAACGACATTTATAA
TGAACGTGAATTGCTCAACAGTATGGGCATTTCGCAGCCTACCGTGGTGTTCGTTTCCAAAAAGGG
GTTGCAAAAAATTTTGAACGTGCAAAAAAAGCTCCCAATCATCCAAAAAATTATTATCATGGATTC
TAAAACGGATTACCAGGGATTTCAGTCGATGTACACGTTCGTCACATCTCATCTACCTCCCGGTTTT
AATGAATACGATTTTGTGCCAGAGTCCTTCGATAGGGACAAGACAATTGCACTGATCATGAACTCC
TCTGGATCTACTGGTCTGCCTAAAGGTGTCGCTCTGCCTCATAGAACTGCCTGCGTGAGATTCTCGC
ATGCCAGAGATCCTATTTTTGGCAATCAAATCATTCCGGATACTGCGATTTTAAGTGTTGTTCCATT
CCATCACGGTTTTGGAATGTTTACTACACTCGGATATTTGATATGTGGATTTCGAGTCGTCTTAATG
TATAGATTTGAAGAAGAGCTGTTTCTGAGGAGCCTTCAGGATTACAAGATTCAAAGTGCGCTGCTG
GTGCCAACCCTATTCTCCTTCTTCGCCAAAAGCACTCTGATTGACAAATACGATTTATCTAATTTAC
ACGAAATTGCTTCTGGTGGCGCTCCCCTCTCTAAGGAAGTCGGGGAAGCGGTTGCCAAGAGGTTCC
ATCTGCCAGGTATCAGGCAAGGATATGGGCTCACTGAGACTACATCAGCTATTCTGATTACACCCG
AGGGGGATGATAAACCGGGCGCGGTCGGTAAAGTTGTTCCATTTTTTGAAGCGAAGGTTGTGGATC
TGGATACCGGGAAAACGCTGGGCGTTAATCAAAGAGGCGAACTGTGTGTGAGAGGTCCTATGATT
ATGTCCGGTTATGTAAACAATCCGGAAGCGACCAACGCCTTGATTGACAAGGATGGATGGCTACAT
TCTGGAGACATAGCTTACTGGGACGAAGACGAACACTTCTTCATCGTTGACCGCCTGAAGTCTCTG
ATTAAGTACAAAGGCTATCAGGTGGCTCCCGCTGAATTGGAATCCATCTTGCTCCAACACCCCAAC
ATCTTCGACGCAGGTGTCGCAGGTCTTCCCGACGATGACGCCGGTGAACTTCCCGCCGCCGTTGTT
GTTTTGGAGCACGGAAAGACGATGACGGAAAAAGAGATCGTGGATTACGTCGCCAGTCAAGTAAC
AACCGCGAAAAAGTTGCGCGGAGGAGTTGTGTTTGTGGACGAAGTACCGAAAGGTCTTACCGGAA
AACTCGACGCAAGAAAAATCAGAGAGATCCTCATAAAGGCCAAGAAGGGCGGAAAGATCGCCGT
G**TAA**gtgcgagaggccgagggccagaacaacatccgcctaccatccatcattgttataaaaaa**CTT**aggaaccaggtccacacagccgccagcccatcaa
ccatccactcccacgattggagcca**ATG**GGGATaTCCGAGGCGATCGTCGACATTCCTGAGATTCCTGGGTTCAAG
GACTTGGAGCCCATGGAGCAGTTCATCGCACAGGTCGATCTGTGTGTGGACTGCACAACTGGCTGC
CTCAAAGGGCTTGCCAACGTGCAGTGTTCTGACCTGCTCAAGAAGTGGCTGCCGCAACGCTGTGCG
ACCTTTGCCAGCAAGATCCAGGGCCAGGTGGACAAGATCAAGGGGGCCGGTGGTGACGGAGGAGG
GGGCTCCGGAgattataaggatgacgatgacCCGCGGTCCGGAGGAGGGGGCTCCCCCatcaCAAGTTTGTACttggt
tgaactccggaaccctaatcctgccctaggtggtGTGAtgcgagaggccgagggccagaacaacatccgcctaccatccatcattgttataaaaaa**CTT**agg
aaccaggtccacacagccgccagcccatcaaccatccactcccacgattGTCAcacccaTTAAAAAGGGGTCTTCACACTCGAAGA
TTTCGTTGGGGACTGGCGACAGACAGCCGGCTACAACCTGGACCAAGTCCTTGAACAGGGAGGTGT
GTCCAGTTTGTTTCAGAATCTCGGGGTGTCCGTAACTCCGATCCAAAGGATTGTCCTGAGCGGTGA
AAATGGGCTGAAGATCGACATCCATGTCATCATCCCGTATGAAGGTCTGAGCGGCGACCAAATGG
GCCAGATCGAAAAAATTTTTAAGGTGGTGTACCCTGTGGATGATCATCACTTTAAGGTGATCCTGC
ACTATGGCACACTGGTAATCGACGGGGTTACGCCGAACATGATCGACTATTTCGGACGGCCGTATG
AAGGCATCGCCGTGTTCGACGGCAAAAAGATCACTGTAACAGGGACCCTGTGGAACGGCAACAAA
ATTATCGACGAGCGCCTGATCAACCCCGACGGCTCCCTGCTGTTCCGAGTAACCATCAACGGAGTG
ACCGGCTGGCGGCTGTGCGAACGCATTCTGGC**GTA**attggttgaactccggaaccctaatcctgccctaggtggttaggcattattt
gcaatatattaaagaaaa**CTT**tgaaaatacgaagtttctattcccagctttgtctggt
